# TRPM6 and TRPM7 differentially contribute to the relief of heteromeric TRPM6/7 channels from inhibition by cytosolic Mg^2+^ and Mg·ATP

**DOI:** 10.1038/s41598-017-08144-1

**Published:** 2017-08-18

**Authors:** Silvia Ferioli, Susanna Zierler, Joanna Zaißerer, Johann Schredelseker, Thomas Gudermann, Vladimir Chubanov

**Affiliations:** 1 Walther Straub Institute of Pharmacology and Toxicology, LMU Munich, Munich, Germany; 2 German Center for Lung Research, Munich, Germany; 3 German Centre for Cardiovascular Research, Munich Heart Alliance, Munich, Germany

## Abstract

TRPM6 and its homologue TRPM7 are α-kinase-coupled divalent cation-selective channels activated upon reduction of cytosolic levels of Mg^2+^ and Mg·ATP. TRPM6 is vital for organismal Mg^2+^ balance. However, mechanistically the cellular role and functional nonredundancy of TRPM6 remain incompletely understood. Comparative analysis of native currents in primary cells from TRPM6- *versus* TRPM7-deficient mice supported the concept that native TRPM6 primarily functions as a constituent of heteromeric TRPM6/7 channels. However, heterologous expression of the human TRPM6 protein engendered controversial results with respect to channel characteristics including its regulation by Mg^2+^ and Mg·ATP. To resolve this issue, we cloned the mouse TRPM6 (mTRPM6) cDNA and compared its functional characteristics to mouse TRPM7 (mTRPM7) after heterologous expression. Notably, we observed that mTRPM6 and mTRPM7 differentially regulate properties of heteromeric mTRPM6/7 channels: In the presence of mTRPM7, the extreme sensitivity of functionally expressed homomeric mTRPM6 to Mg^2+^ is tuned to higher concentrations, whereas mTRPM6 relieves mTRPM7 from the tight inhibition by Mg·ATP. Consequently, the association of mTRPM6 with mTRPM7 allows for high constitutive activity of mTRPM6/7 in the presence of physiological levels of Mg^2+^ and Mg·ATP, thus laying the mechanistic foundation for constant vectorial Mg^2+^ transport specifically into epithelial cells.

## Introduction

Transient receptor potential melastatin 6 (TRPM6) and 7 (TRPM7) are extraordinary proteins comprising two distinct functional moieties: an ion channel segment and an α-type serine/threonine kinase domain^1, 2^. Experiments with TRPM7*-*deficient cell lines revealed that TRPM7 regulates salient cell processes such as Mg^2+^ metabolism^3–5^, Ca^2+^ signaling^6–8^, chromatin modification^9^, cell motility^10–13^, proliferation^6–8, 14–16^ and differentiation^17–19^. Genetic inactivation of *Trpm7* in mice results in early embryonic death^17, 19^. Conditional tissue-specific inactivation of *Trpm7* in mice showed that TRPM7 plays a critical role in morphogenesis of various internal organs^19–21^. In contrast to global *Trpm7* null mutations, specific genetic inactivation of the kinase activity (referred to a ‘kinase-dead’ *Trpm7* mutation) does not impede embryonic development^22^. Adult ‘kinase-dead’ *Trpm7* mice are more resistant to dietary Mg^2+^ deprivation in terms of survival and develop a mild form of organismal Mg^2+^ deficiency^23^. These results were interpreted to mean that the TRPM7 kinase moiety is necessary for an adaptive systemic response to Mg^2+^ deficiency^23^. Recently, a search for genetic forms of macrothrombocytopenia in humans resulted in the discovery of two pedigrees harbouring loss-of-function point mutations in the human *TRPM7* gene^24^. These patients presented with impaired thrombopoiesis due to altered cellular Mg^2+^ homeostasis and cytoskeletal architecture^24^.

Recombinant TRPM7 proteins from several vertebrate species have been cloned and functionally characterized^2^. With minor exceptions^25^, key channel properties of TRPM7 orthologues were found to be highly conserved. Briefly, recombinant TRPM7 forms homotetrameric channel complexes highly permeable to Ca^2+^, Mg^2+^ and Zn^2+^ ^15, 26–28^. Intracellular and extracellular Mg^2+^ regulates TRPM7 channel activity. External Mg^2+^ acts as a permeant blocker of the channel pore^15, 26–29^. Internally applied Mg^2+^ ([Mg^2+^]_i_) and Mg·ATP ([Mg·ATP]_i_) inhibit TRPM7 currents^15^. Accordingly, cytosolic concentrations of Mg·ATP and free Mg^2+^ have been suggested as key feedback regulators of TRPM7 function implying that depletion of intracellular Mg^2+^ and Mg·ATP promotes TRPM7-mediated uptake of extracellular Mg^2+^ ^15^. TRPM7 is assumed to be a ubiquitously expressed protein^2^, and endogenous TRPM7 currents, referred to as magnesium nucleotide-regulated metal ion currents (MagNuM)^15, 30^ and magnesium-inhibited cation currents (MIC)^31^, have been detected in all cell types examined so far^2^.

Loss-of-function mutations in the human *TRPM6* gene give rise to autosomal recessive hypomagnesemia, also called *primary hypomagnesemia type 1*, *intestinal* (HOMG1) or *hypomagnesemia with secondary hypocalcemia* (HSH)^32–35^. HSH manifests in early infancy with 3- to 10-fold decreased serum Mg^2+^ concentrations, generalized convulsions and muscle spasms. Relief of clinical symptoms can be achieved by administration of high doses of Mg^2+^ ^32–35^. Unexpectedly, *Trpm6* null mice die at embryonic day 12.5 (e12.5)^36^. Recently, our group employed a set of newly generated mouse strains to define the *in vivo* role of TRPM6^37^. We showed that TRPM6 activity in extraembryonic cells of the placenta and yolk sac is essential for embryonic survival of mice^37^. In adult mice, TRPM6 is required in intestinal epithelial cells to maintain organismal Mg^2+^ balance^37^. Thus, TRPM6 is a central gatekeeper of organismal Mg^2+^ balance in mammals, and its role cannot be compensated by any other channel such as TRPM7. Against the backdrop of the Mg^2+^-permeable ubiquitously expressed TRPM7, the pivotal role of TRPM6 in transporting epithelia is only incompletely understood.

In contrast to the situation with TRPM7, functional characterization of the TRPM6 channel for the most part relies on experiments with the human TRPM6 (hTRPM6) cDNA. Our group^38, 39^ and other investigators^40, 41^ observed that hTRPM6 does not efficiently form homomultimeric channels in the plasma membrane, but requires TRPM7 to be co-targeted to the cell surface^38, 39^. Within heteromeric channel complexes (referred to TRPM6/7 channels), hTRPM6 increases the current amplitude of TRPM6/7 heteromers as compared to TRPM7 homomers^38, 39^. According to other authors^42, 43^, overexpressed hTRPM6 was able to form homomeric channels with biophysical characteristics resembling those of TRPM7 including the high channel pore selectivity for divalent cations and regulation by [Mg^2+^]_i_ and [Mg·ATP]_i_. More recently, it has been reported that expression of recombinant hTRPM6 gives rise to functional channels only if the hTRPM6 cDNA was inserted into the pCINeo-IRES-GFP vector (referred to pCINeo-hTRPM6-IRES-GFP), whereas the same cDNA sequence placed in various other expression plasmids did not yield functional hTRPM6 channels^27^. This feature of hTRPM6 appears to be unique among TRP proteins, and is still lacking any mechanistic explanation. Furthermore, different researchers used the same pCINeo-hTRPM6-IRES-GFP construct to report contradictory results regarding the sensitivity of hTRPM6 to [Mg·ATP]_i_, ranging from full suppression in the presence of physiological levels of [Mg·ATP]_i_^44^ to complete insensitivity^27^. Hence, the functional characteristics of recombinant TRPM6 require further clarification.

In light of the latter controversy, we opted for the functional analysis of primary cells lacking the endogenous mouse TRPM6 (mTRPM6) protein rather than for heterologous expression models and employed trophoblast stem (TS) cells derived from *Trpm6*- and *Trpm7*-gene deficient mouse blastocysts^37^. We found that wildtype TS cells express both mTRPM6 and mTRPM7, thus mirroring the *in vivo* situation in transporting epithelial cells invariably co-expressing both proteins. We showed that wildtype TS cells exhibit TRPM7-like currents, and that genetic inactivation of mTRPM6 reduces the amplitude of these currents. Ionic currents in mTRPM6-deficient TS cells were substantially more sensitive to [Mg·ATP]_i_ but equally affected by [Mg^2+^]_i_^37^. In contrast, deletion of mTRPM7 resulted in complete disappearance of TRPM7-like currents in TS cells^37^. These findings support the concept that native TRPM6 primarily functions as a subunit of heteromeric TRPM6/7 channels and that [Mg·ATP]_i_ most likely is a crucial endogenous regulator of Mg^2+^ uptake mediated by TRPM6/7 channels.

In the present paper we investigated the role of channel and kinase domains of TRPM6 for the sensitivity of TRPM6/7 channels to [Mg^2+^]_i_ and [Mg·ATP]_i_. Because the functional consequences of the heterologous expression of hTRPM6 are surrounded by considerable controversy, we cloned and functionally characterized mTRPM6. Our experiments show that the functional hallmarks of recombinant mTRPM6 in HEK 293 cells recapitulate key findings that emerged from the comparative assessment of mTRPM6- and mTRPM7-deficient TS stem cells^37^, lending credence to the concept that the association of mTRPM6 with mTRPM7 substantially alters the regulatory properties of the mTRPM6/7 channels such as their sensitivity to cytosolic Mg·ATP. As a consequence, mTRPM6/7 channels display the unique property of remaining constitutively active in the presence of physiological levels of cytosolic Mg^2+^ and Mg·ATP. Hence, the association of mTRPM6 with mTRPM7 will likely lead to a constant Mg^2+^ uptake, thus providing a mechanistic explanation for efficient Mg^2+^ transport into epithelial cells only in the presence of mTRPM6.

## Results

### Cloning and functional expression of recombinant mTRPM6 cDNA

**Figure 1:**
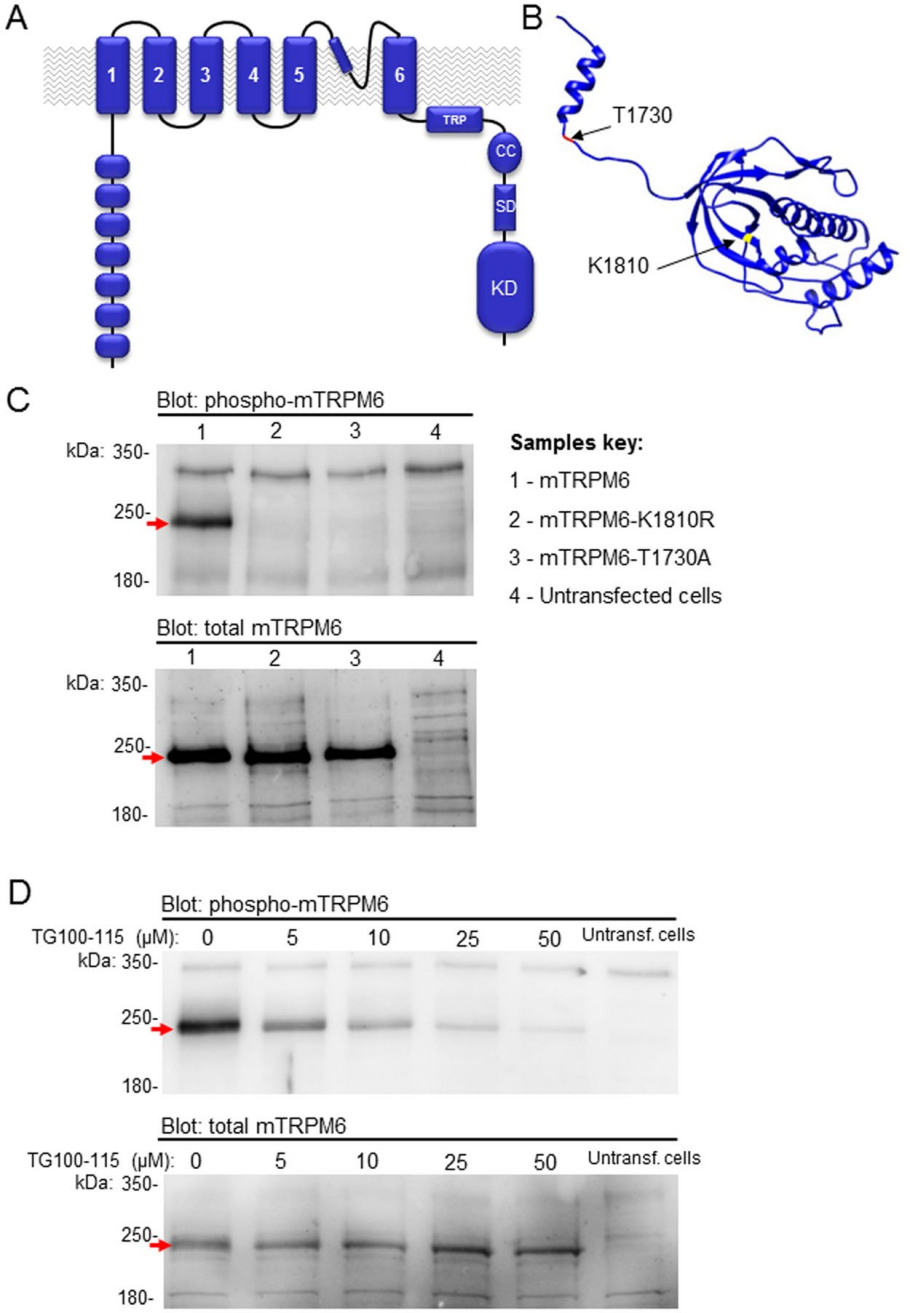
Domain topology of the cloned murine kinase-coupled channel TRPM6. (**A**) The plasma membrane channel segment of mTRPM6 comprises six transmembrane helices (*1–6*). A short stretch between the 5th and 6th helices contains a predicted pore forming loop and a pore helix. A N-terminus of mTRPM6 contains a set of predicted ankyrin-like repeats^1^. The mTRPM6 C-terminus contains a highly conserved transient receptor potential (*TRP*) domain, a coiled-coil (*CC*) domain, a kinase substrate domain (*SD*) and a kinase domain (*KD*). (**B**) 3D model of the mTRPM6 kinase domain generated as described previously^1^ using annotated coordinates of the mouse TRPM7 kinase (PDB code 1IA9^46^). K1810 is a highly conserved residue located in the catalytic site of the kinase domain. T1730 is a residue subjected to autophosphorylation by the mTRPM6 kinase. (**C**) Representative Western blot analysis of mTRPM6 variants using the anti-(p)T1730 mTRPM6 antibody (*upper* panel) followed by stripping and re-probing of total mTRPM6 expression levels by anti-mTRPM6 polyclonal ab47017 antibody (*lower* panel). The experiment was repeated three times with similar results. (**D**) Effect of TG100–115 on the autophosphorylation of mTRPM6. HEK 293 cells were transiently transfected with mTRPM6. 24 h after transfection, the indicated concentrations of TG100–115 were added to the cell culture medium and cells were cultured for additional 12 h and immunoreactivity of mTRPM6 was probed as in (**C**). A representative Western blot is shown. The experiment was repeated two times with similar results.

*Mus musculus* is broadly used as an *in vivo* experimental model to study the physiological role of ion channels including TRPM6 and TRPM7. Surprisingly, the murine TRPM6 protein has not been investigated so far in heterologous expression systems. To resolve the discrepancies obtained with heterologous expression of human TRPM6, we cloned the mouse TRPM6 cDNA into pIRES2-EGFP vector and assessed its channel and kinase activities upon transient expression in HEK 293 cells. We generated a full-length mTRPM6 cDNA (NCBI accession KX375810) using RNA extracted from lung tissue. Sequencing confirmed that the obtained mTRPM6 cDNA contains an open reading frame (ORF) encoding a 2028-amino acid protein, showing 100% protein sequence identity to an NCBI predicted sequence NM_153417.1. Similar to hTRPM6^45^, mTRPM6 comprises a highly conserved N-terminal TRPM domain and a typical TRP-like channel segment followed by a C-terminal kinase moiety (Fig. 1A). We used a previously reported crystal structure of the mTRPM7 kinase^46^ to generate a 3D model of the mTRPM6 kinase domain and the location of residues critical for the catalytic activity of the TRPM6 kinase such as K1810 (K1646 in mTRPM7) (Fig. 1B)^3, 45^. In addition, we predicted a residue that is likely to be autophosphorylated in mTRPM6 such as T1730 (S1567 in mTRPM7^47^) (Fig. 1B).

To evaluate these predictions functionally, we generated mTRPM6 cDNA variants carrying a ‘kinase-dead’ point mutation (mTRPM6-K1810R) and a variant lacking the predicted autophosphorylation site (mTRPM6-T1730A). Next, we transiently expressed wildtype mTRPM6, mTRPM6-K1810R and mTRPM6-T1730A constructs in HEK 293 cells and assessed the expression levels of the recombinant proteins by Western blot analysis (Fig. 1C). In these experiments, we used a rabbit polyclonal antibody designed as a specific probe for mTRPM6 phosphorylated at T1730 ((p)T1730-specific antibody), and a guinea pig anti-TRPM6 polyclonal (ab47017) antibody to identify all mTRPM6 variants. We observed that the ab47017 antibody detected comparable protein expression of wildtype mTRPM6, mTRPM6-K1810R and mTRPM6-T1730A and that mTRPM6-specific bands were of the expected size (232 kDa) (Fig. 1C). The (p)T1730-specific antibody only detected wildtype mTRPM6, but not the mutant variants (Fig. 1C) consistent with the notion that the kinase domain autophosphorylates the mTRPM6 protein at position T1730 and that the K1810R mutation ablates the catalytic activity of mTRPM6 kinase. Consequently, we asked whether the catalytic activity of mTRPM6 kinase can be manipulated in living cells by pharmacological agents. Previously, Davis *et al*.^48^ examined 442 kinases to assess the overall selectivity of 72 well-defined kinase inhibitors. The authors reported that TG100-115 inactivated the purified kinase domain of hTRPM6^48^. We observed that a 12-h culture of mTRPM6-transfected HEK 293 cells in the presence of TG100-115 led to suppression of (p)T1730 immunoreactivity (Fig. 1D). Thus, both the K1810R mutation and TG100-115 enable to block mTRPM6 kinase.

### Assessment of mTRPM6 currents in HEK 293 cells and in trophoblast stem cells

**Figure 2:**
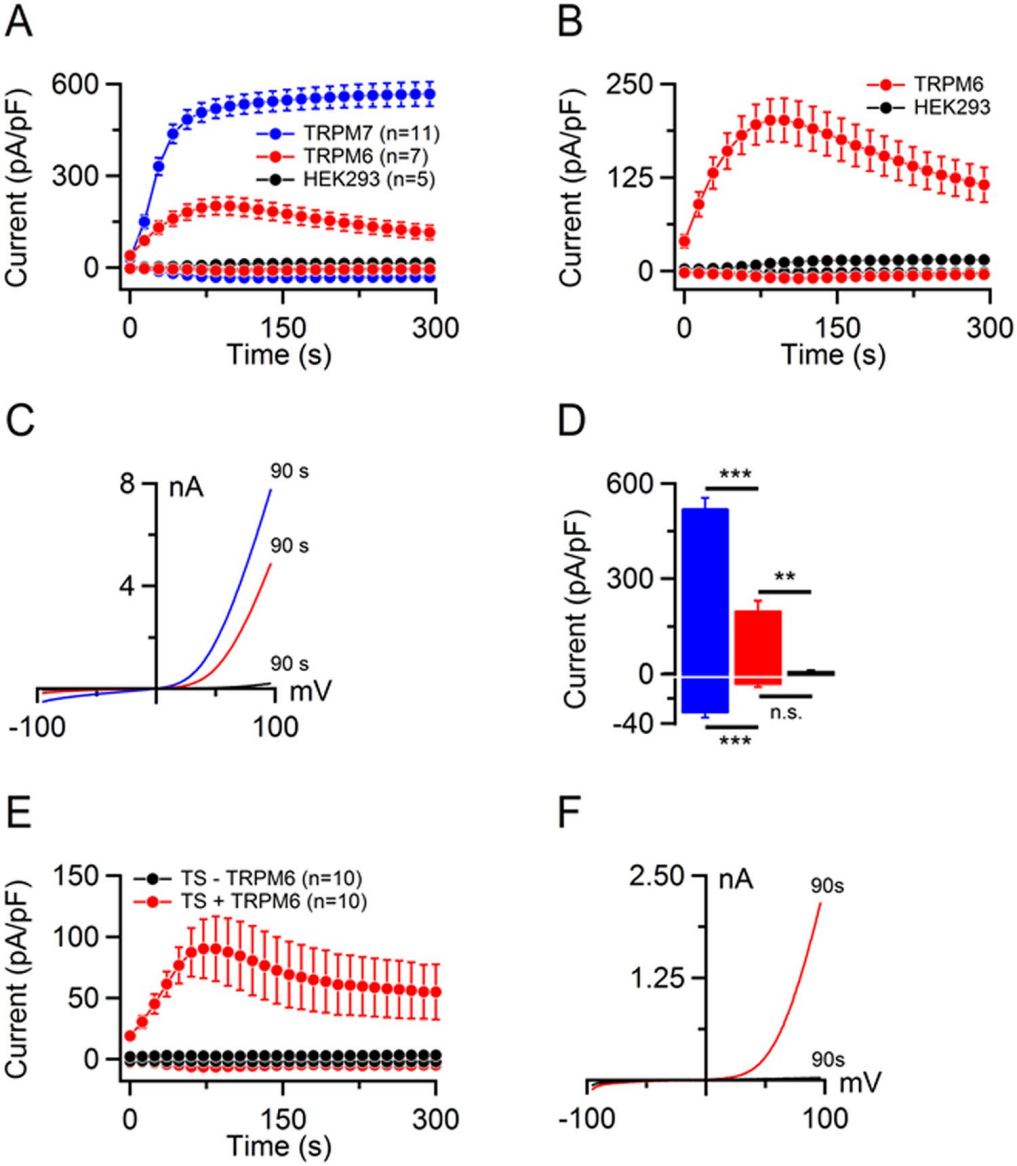
Assessment of mTRPM6 and mTRPM7 currents in HEK 293 and TS cells. (**A**) Whole-cell currents measured in mTRPM6 (red), mTRPM7-transfected (blue) and untransfected (black) HEK 293 cells. Current amplitudes (mean ± SEM) were acquired at −80 and +80 mV and plotted over time. (**B**) Magnification of mTRPM6 and endogenous TRPM7 currents shown in (A). (**C**) Representative current-voltage (I-V) relationships of currents (at 90 s) illustrated in (A). (**D**) Bar graphs of outward (*Upper* panel) and inward (*Lower* panel) currents (mean ± SEM) shown in (A) at 90 s. n, number of cells measured; n.s., not significant; **P < 0.01; ***P < 0.001 (ANOVA). (**E**,**F**) Functional expression of mTRPM6 in trophoblast stem (TS) cells. *Trpm7*-gene deficient TS cells were electroporated with mTRPM6 cDNA and examined as described in (**A**). (**E**) Whole-cell currents (mean ± SEM) measured in mTRPM6-transfected (red) and untransfected (black) TS cells. (**F**) I-V relationships of the currents (at 90 s) shown in (**E**). n, number of cells measured.

HEK 293 cells were transiently transfected with either mTRPM6 or mTRPM7 cDNAs inserted into the pIRES2-EGFP expression vector and EGFP-positive cells were examined by patch-clamp analysis. Whole-cell currents were elicited by a voltage ramp protocol ranging from −100 to +100 mV and a Mg^2+^-free internal solution (Fig. 2A). In HEK 293 cells expressing mTRPM7, outward and inward current amplitudes were small immediately after establishing the whole-cell configuration, but rapidly increased presumably due to depletion of intracellular Mg^2+^. mTRPM7 currents reached a plateau after ~100 s (Fig. 2A). The current-voltage (I-V) relationships of mTRPM7 currents exhibited characteristic features such as small inward and large outward currents with a pronounced rectification, and a reversal potential of about 0 mV (Fig. 2C). Notably, mTRPM6-expressing cells also developed inward and outward currents (Fig. 2A) with an I-V relationship resembling that of mTRPM7 (Fig. 2C). However, other biophysical characteristics of mTRPM6 currents were distinct: While mTRPM6 currents reached a peak at ~100 s of recording, they gradually declined thereafter presumably due to an intrinsic inactivation mechanism (Fig. 2B). Furthermore, we noted that fully developed outward and inward mTRPM6 currents were substantially (~3-fold) smaller when compared to values obtained with mTRPM7 (Fig. 2D).

It has been shown that the endogenous hTRPM7 protein is expressed in HEK 293 cells, and that native TRPM7-like currents can be measured in this cell line^15, 49^. Potentially, the native hTRPM7 protein may form heteromeric channel complexes with recombinant mTRPM6 and thus contaminate our recordings. Indeed, we were able to elicit mTRPM7-like currents in untransfected HEK 293 cells (Fig. 2A,B). However, amplitudes of these endogenous currents were ~10-fold smaller than those in mTRPM6-expressing HEK 293 cells (Fig. 2D), suggesting that the currents measured in mTRPM6-transfected cells could not solely be attributed to the activity of endogenous hTRPM7.

Next, we asked whether mTRPM6 channel activity could be measured in the cells deficient in mTRPM7. To address this question, we used recently generated trophoblast stem (TS) cells derived from *Trpm7* null mouse blastocysts^37^. As reported previously^37^, *Trpm7*-deficient TS cells completely lack native TRPM7-like currents (Fig. 2E,F). We found that transient overexpression of mTRPM6 resulted in whole-cell currents similar to mTRPM6 currents attained in HEK 293 cells. Hence, upon transient overexpression of recombinant protein, activity of homomeric mTRPM6 channel was measurable independently of the presence of endogenous TRPM7 channels.

It has been reported^27^ that transient expression of human TRPM6 (hTRPM6) gives rise to a functional channel only if hTRPM6 cDNA is inserted into a specific expression vector, whereas the same cDNA clone placed in other expression plasmids did not yield functional hTRPM6 channels^27^. We could fully recapitulate this finding (Supplementary Fig. S1). Thus, in analogy to the previous study^27^, transient transfection of the pCINeo-hTRPM6-IRES-GFP expression construct allowed us to detect hTRPM6 currents, well comparable to mTRPM6 currents (Supplementary Fig. S1A). However, when investigating a HEK 293 T-REx cell line stably expressing hTRPM6^27, 50^, we found that doxycycline-induced cells displayed currents undistinguishable from endogenous currents measured in uninduced cells (Supplementary Fig. S1B). Consequently, we asked whether functional expression of mTRPM6 cDNA would also be dependent on the expression system employed. We subcloned the mTRPM6 cDNA into the frequently used pcDNA3.1 vector^38^ and co-transfected this construct with a small amount of EGFP cDNA into HEK 293 cells. Patch-clamp analysis of EGFP-positive HEK 293 cells (Supplementary Fig. 2A) revealed currents very similar to those detected in cells transfected with pIRES2-mTRPM6-EGFP (Fig. 2B), suggesting that expression vectors are interchangeable for functional assessment of the mTRPM6 cDNA. In our follow-up experiments we used the pIRES2-mTRPM6-EGFP and pIRES2-mTRPM7-EGFP constructs.

### Cation permeability of mTRPM6

**Figure 3:**
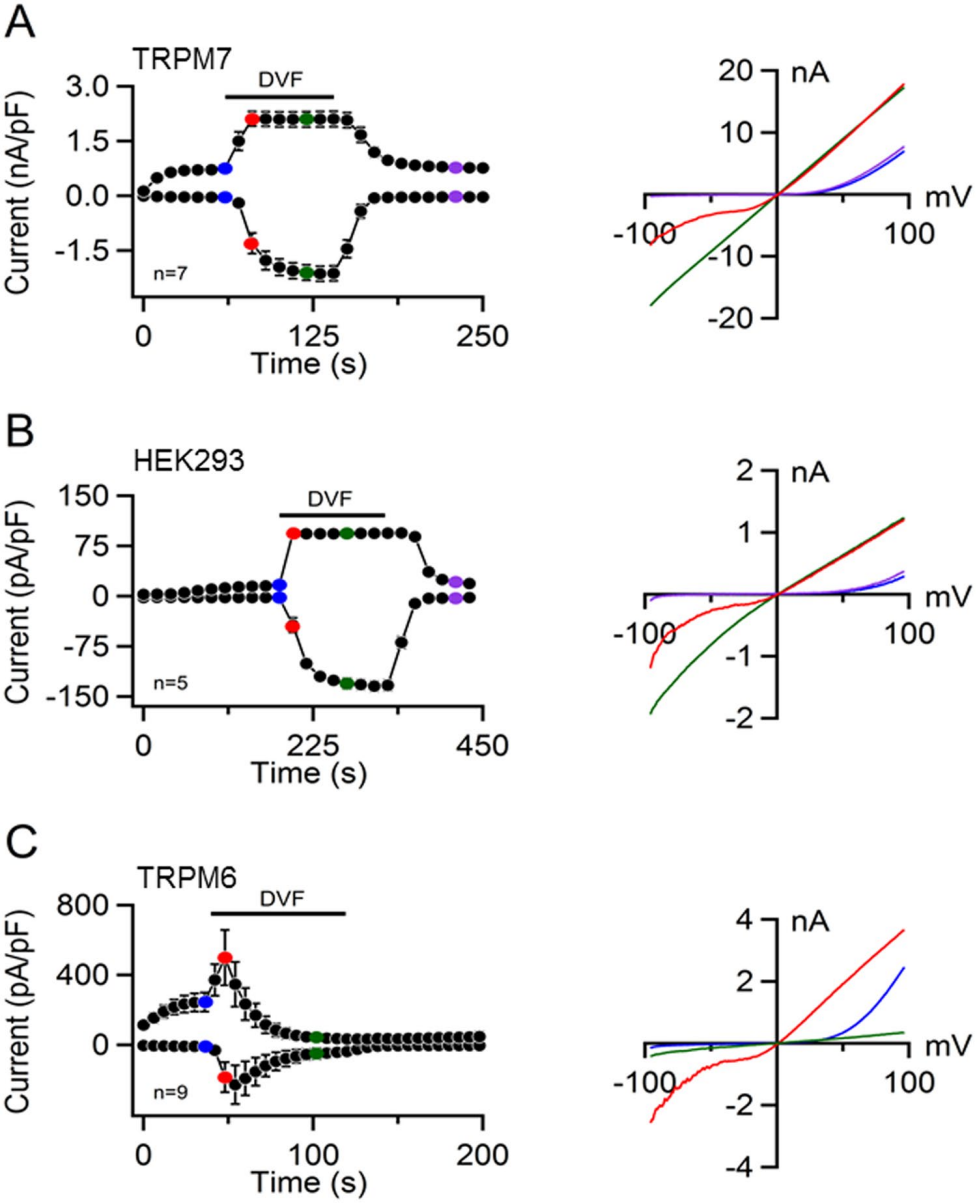
Assessment of mTRPM6 and mTRPM7 currents in a divalent cation-free (DVF) extracellular solution. Whole-cell currents measured in mTRPM7-transfected (**A**), untransfected (**B**) and mTRPM6-transfected HEK 293 cells (**C**). *Left panels*: Current amplitudes (mean ± SEM) were measured at −80 and +80 mV and plotted over time. Currents were induced using the standard [Mg^2+^]_i_-free intracellular solution and the standard external solution. When currents were fully activated, cells were perfused with the DVF solution as indicated by the black bars. *Right panels*: Representative I-V relationships obtained from individual ramps before (blue), during (red and green) and after (violet) DVF application as indicated in the *Left panels* by coloured data points. n, number of cells measured.

TRPM7 channel is highly permeable to a range of divalent cations including Ca^2+^, Mg^2+^ and Zn^2+^ ^15, 26^. Therefore, we asked whether the cation permeation profile of mTRPM6 differs from that of mTRPM7. In a first set of experiments, we assessed the permeation block of mTRPM7 currents by extracellular divalent cations, a well-known characteristic feature of TRPM7^15, 26^. As expected, exposure of mTRPM7-expressing cells to a divalent cation-free (DVF) solution entailed large monovalent cation currents with a characteristic linear I-V relationship (Fig. 3A). These monovalent mTRPM7 currents were stable over the whole time of exposure. After wash-out, this characteristic response of mTRPM7 was fully reversed (Fig. 3A). We also noted that the endogenous TRPM7-like currents reliably recapitulated functional properties of recombinant mTRPM7 (Fig. 3B). In contrast, mTRPM6-transfected cells only showed an initial TRPM7-like response to the application of DVF solution, characterized by prompt increases of outward and inward currents (red traces; Fig. 3C) followed by a fast, irreversible inactivation of mTRPM6 activity (green traces; Fig. 3C). Hence, unlike the situation with mTRPM7, the removal of external divalent cations relieved the mTRPM6 channel from the divalent cation permeation block followed by the fast rundown of channel activity.

**Figure 4:**
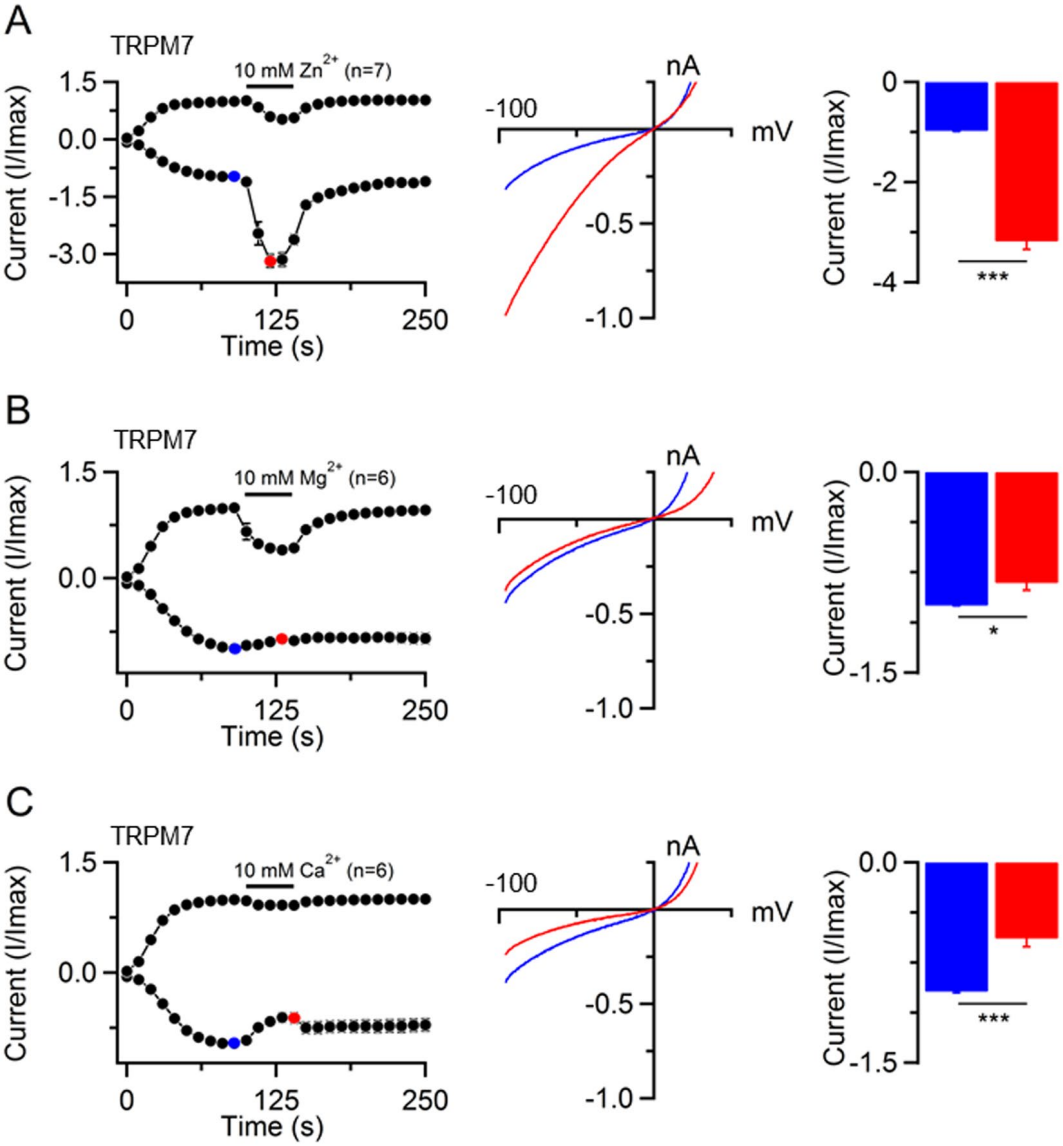
Examining of divalent cations permeability of mTRPM7. (**A**) *Left panel:* Whole-cell currents were recorded in mTRPM7-transfected HEK 293 cells using the standard [Mg^2+^]_i_-free internal solution and standard external solution. When currents started to develop, cells were subsequently exposed to the external solution containing 10 mM Zn^2+^ as indicated by a bar. Data are shown as I/Imax ± SEM (Imax value was obtained in a ramp before application of 10 mM Zn^2+^). *Middle panel*: Representative I-V relationships of inward currents obtained before (blue) and during (red) application of 10 mM Zn^2+^ as indicated in the *Left panel*. *Right panel*: Bar graphs of inward currents (−80 mV, mean I/Imax ± SEM) obtained before (blue) and during (red) application of 10 mM Zn^2+^ as indicated in the *Left panel*. (**B**,**C**) Changes in the inward mTRPM7 currents by exposure of cells to external solutions containing 10 mM Mg^2+^ (**B**) and 10 mM Ca^2+^ (**C**). Measurements were performed similarly to (**A**). n, number of cells measured; *P < 0.05; ***P < 0.001 (two-tailed t-test).

**Figure 5:**
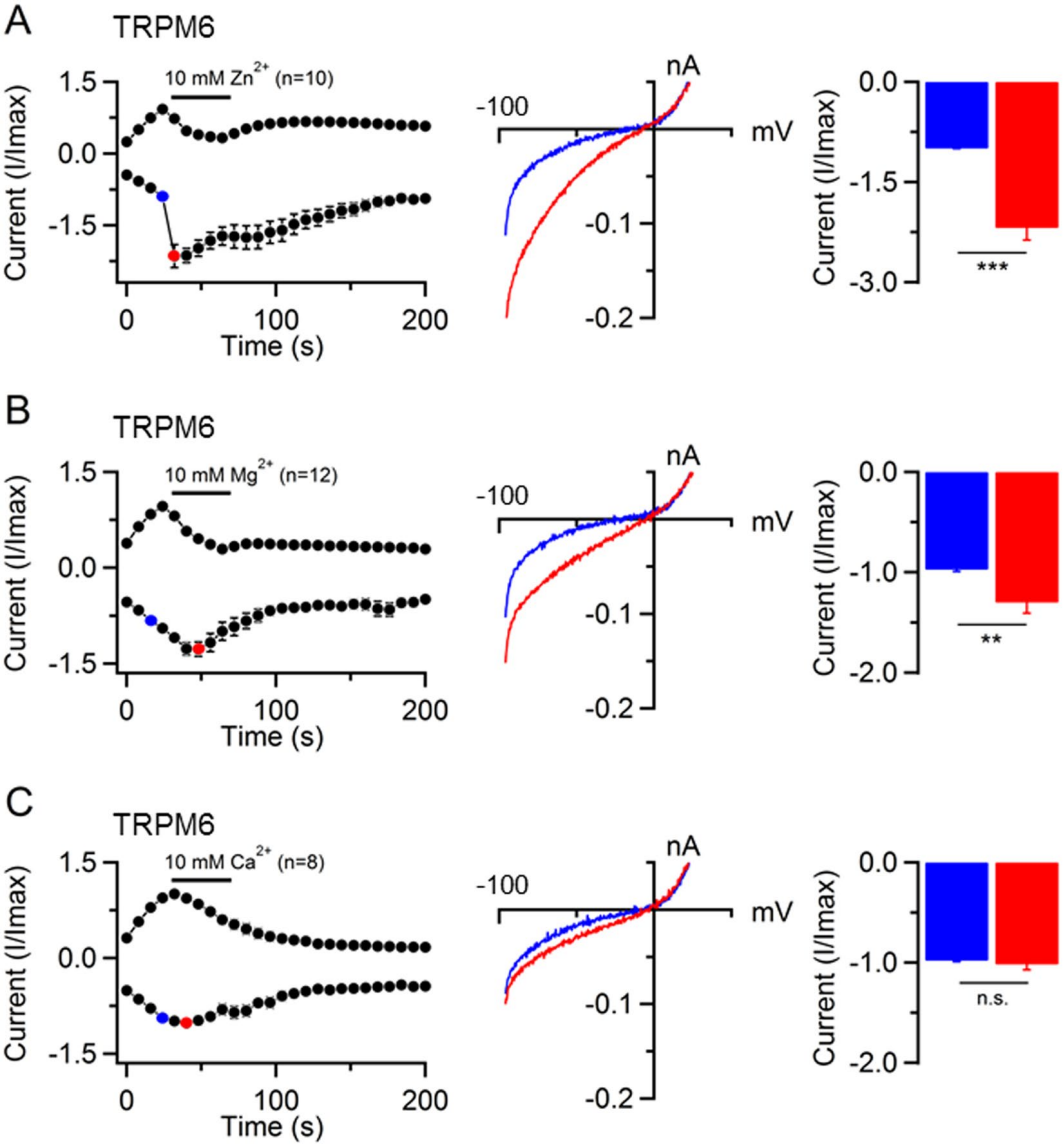
Determination of divalent cations permeability of mTRPM6. Measurements were performed similarly to Fig. 4. (**A**) *Left panel:* Whole-cell currents were recorded in mTRPM6-transfected HEK 293 cells using the standard [Mg^2+^]_i_-free internal solution and standard external saline. When currents started to develop, the cells were exposed to an external solution containing 10 mM Zn^2+^ as indicated by a bar. Data are shown as I/Imax ± SEM (Imax value was obtained in a ramp before application of 10 mM Zn^2+^). *Middle panel*: Representative I-V relationships of inward currents obtained before (blue) and during (red) application of 10 mM Zn^2+^ as indicated in the *Left panel*. *Right panel*: Bar graphs of inward currents (−80 mV, mean I/Imax ± SEM) obtained before (blue) and during (red) application of 10 mM Zn^2+^ as indicated in the *Left panels*. (**B**,**C**) Changes in the inward mTRPM6 currents by exposure of cells to external solutions containing either 10 mM Mg^2+^ (**B**) or 10 mM Ca^2+^ (**C**). n, number of cells measured; n.s., not significant; **P < 0.01; ***P < 0.001 (two-tailed t-test).

Divalent cation selectivity of TRPM7 has been determined by two means: (i) by shifts in the reversal potential of monovalent TRPM7 currents to more positive values after application of external solutions containing individual divalent cations^15, 26, 29^, and (ii) by changes of inward currents in cells exposed to external solutions containing only divalent cations^15, 26, 29^. Since a reliable assessment of the reversal potential of monovalent mTRPM6 currents was not possible (Fig. 3C), we used the second option as outlined in Fig. 4 and Fig. 5. We induced mTRPM7 and mTRPM6 currents using standard internal and external solutions containing 1 mM CaCl_2_ and 2 mM MgCl_2_. When currents were developed, cells were exposed to external solutions containing 10 mM of individual specific divalent cations (Zn^2+^ is poorly soluble above 10 mM at pH 7.0). To prevent Na^+^ from passing through the channels along with divalent cations, external monovalent cations were replaced by the non-permeant N-methyl-D-glucamine (NMDG). Similar to a previous study^26^, the exposure of mTRPM7-transfected cells to 10 mM Zn^2+^ caused a ~3-fold increase in inward currents (Fig. 4A). The perfusion of cells with 10 mM Mg^2+^ or 10 mM Ca^2+^ led to a modest, but statistically significant reduction of inward currents (Fig. 4B,C). Thus, in accord with previous publications^15, 26, 29^, mTRPM7 showed a higher permeability for Zn^2+^ as compared to Mg^2+^ and Ca^2+^ under these experimental conditions. Corresponding experiments with mTRPM6 expressing cells showed that mTRPM6 is also highly permeable to Zn^2+^ as the application of Zn^2+^-based external solution caused a ~2-fold increase of inward currents (Fig. 5A). Unlike mTRPM7, however, inward currents of mTRPM6 increased upon exposure of 10 mM Mg^2+^ (Fig. 5B) and were unaltered in the presence of 10 mM Ca^2+^ (Fig. 5C) indicating that the mTRPM6 channel is more permeable to Mg^2+^ as compared to Ca^2+^.

Finally, we tested whether mTRPM6 is also permeable to nonabundant trace cations such as Ba^2+^ (Supplementary Fig. S3). In line with a previous study^26^, we observed that the exposure of mTRPM7-transfected cells to 10 mM Ba^2+^ caused an increase in outward and inward currents (Supplementary Fig. S3A). In contrast, only outward currents of mTRPM6 were elevated in the presence of 10 mM Ba^2+^ (Supplementary Fig. S3B), suggesting that the mTRPM6 channel is less permeable to Ba^2+^ as compared to mTRPM7. To summarize, our experiments show that the divalent cation permeation profile of mTRPM6 is similar, but not identical to that of mTRPM7.

### High sensitivity of mTRPM6 currents to intracellular Mg^2+^

**Figure 6:**
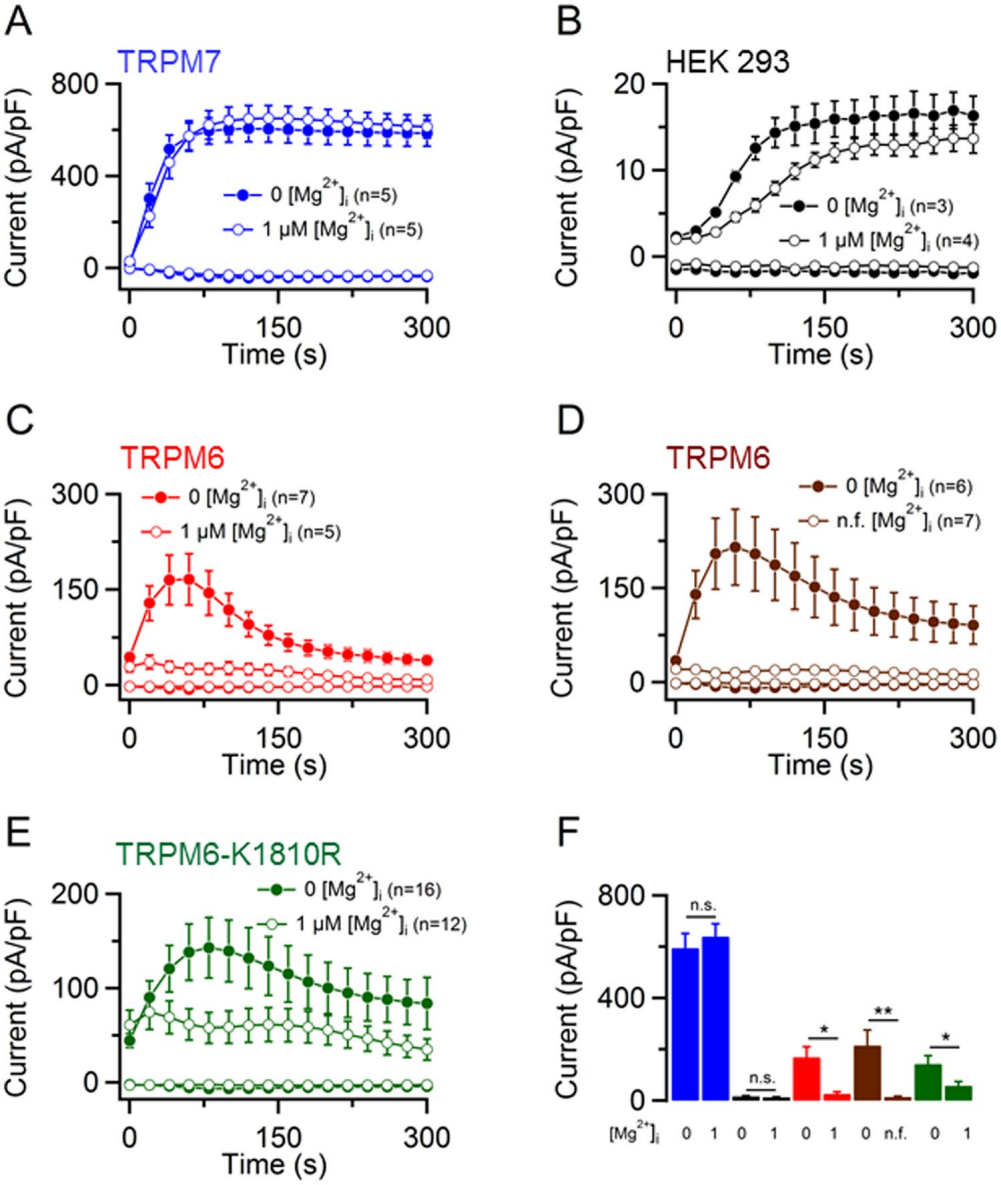
Effects of cytosolic Mg^2+^ on mTRPM6 and mTRPM7 currents. Whole-cell currents (mean ± SEM) measured in mTRPM7-transfected HEK 293 cells (**A**), untransfected HEK 293 cells (**B**), or cells transfected either by wildtype mTRPM6 (**C**,**D**) or ‘kinase-dead’ mTRPM6-K1810R variant (**E**). In (**A**–**C** and **E**) cells were perfused either with a standard [Mg^2+^]_i_-free intracellular solution or with a solution containing 1 µM free [Mg^2+^]_i_. In (**D**) measurements were performed with mTRPM6-transfected cells as in (**C**) except that a nominally [Mg^2+^]_i_-free solution (*n*.*f*.) was used. (**F**) Bar graphs of outward currents (−80 mV) shown in (**A**–**E**). Current amplitudes (mean ± SEM) were extracted at time intervals when the currents were maximally induced: at 200 s for mTRPM7 (blue) and untransfected cells (black), 50 s for wildtype mTRPM6 (red and brown), 80 s for mTRPM6-K1810R (green). n, number of cells measured; n.s., not significant; *P < 0.05; **P < 0.01 (two-tailed t-test).

A remarkable feature of the TRPM7 channel is its high sensitivity to physiological levels of cytosolic Mg^2+^ ([Mg^2+^]_i_) and Mg·ATP ([Mg·ATP]_i_)^15, 26, 27, 51^. Therefore, we compared the effects of [Mg^2+^]_i_ and [Mg·ATP]_i_ on mTRPM6 and mTRPM7 currents using solutions containing various concentrations of free Mg^2+^ and Mg·ATP. In initial experiments, we studied mTRPM7 currents in the presence of 1 µM free [Mg^2+^]_i_ (Fig. 6A,F). In line with previous reports^15, 26, 27, 51^, we observed no inhibitory effect of 1 µM free [Mg^2+^]_i_ on mTRPM7 (Fig. 6A,F) and only a moderate suppression of endogenous TRPM7-like currents (Fig. 6B,F). Surprisingly, we found that currents in mTRPM6-transfected cells did not develop at all in the presence of 1 µM [Mg^2+^]_i_ (Fig. 6C,F). Consistently, mTRPM6 overexpressed in mTRPM7-deficient TS cells was also inactive in the presence of 1 µM free [Mg^2+^]_i_ (Supplementary Fig. S2B). Furthermore, even when we applied a nominally [Mg^2+^]_i_-free intracellular solution (Fig. 6D,F), cells transfected with mTRPM6 cDNA failed to develop any currents indicating that mere traces of free Mg^2+^ are sufficient to block mTRPM6. Such unexpected sensitivity of mTRPM6 to Mg^2+^ prompted us to examine the influence of 1 µM [Mg^2+^]_i_ on currents in HEK 293 cells transfected with pCINeo-hTRPM6-IRES-GFP (Supplementary Fig. S1A). We found that hTRPM6 currents were not affected by 1 µM [Mg^2+^]_i_ (Supplementary Fig. S1A), indicating that the high sensitivity of mTRPM6 to cytosolic Mg^2+^ can be attributed to the mTRPM6 channel itself rather than to the experimental conditions used. Since known Mg^2+^ chelators, such as EDTA or EGTA, do not allow to reliably prepare a saline solution containing [Mg^2+^]_i_ in the nM range, we did not attempt to obtain concentration-response data for mTRPM6 currents.

It has been suggested that the catalytic activity of the TRPM7 kinase domain regulates the sensitivity of the TRPM7 channel to [Mg^2+^]_i_ and [Mg·ATP]_i_^51^. Therefore, we asked whether the kinase activity of mTRPM6 might be responsible for the high sensitivity of mTRPM6 to [Mg^2+^]_i_. To address this question, we examined cells transiently expressing the ‘kinase-dead’ mTRPM6-K1810R variant (Fig. 1B) and found that mTRPM6-K1810R remained inactive in the presence of 1 µM [Mg^2+^]_i_, but could be induced using the standard [Mg^2+^]_i_-free intracellular pipette solution (Fig. 6E,F). We conclude that the kinase activity of mTRPM6 is not involved in the strong inhibitory effect of [Mg^2+^]_i_ on the mTRPM6 channel activity.

**Figure 7:**
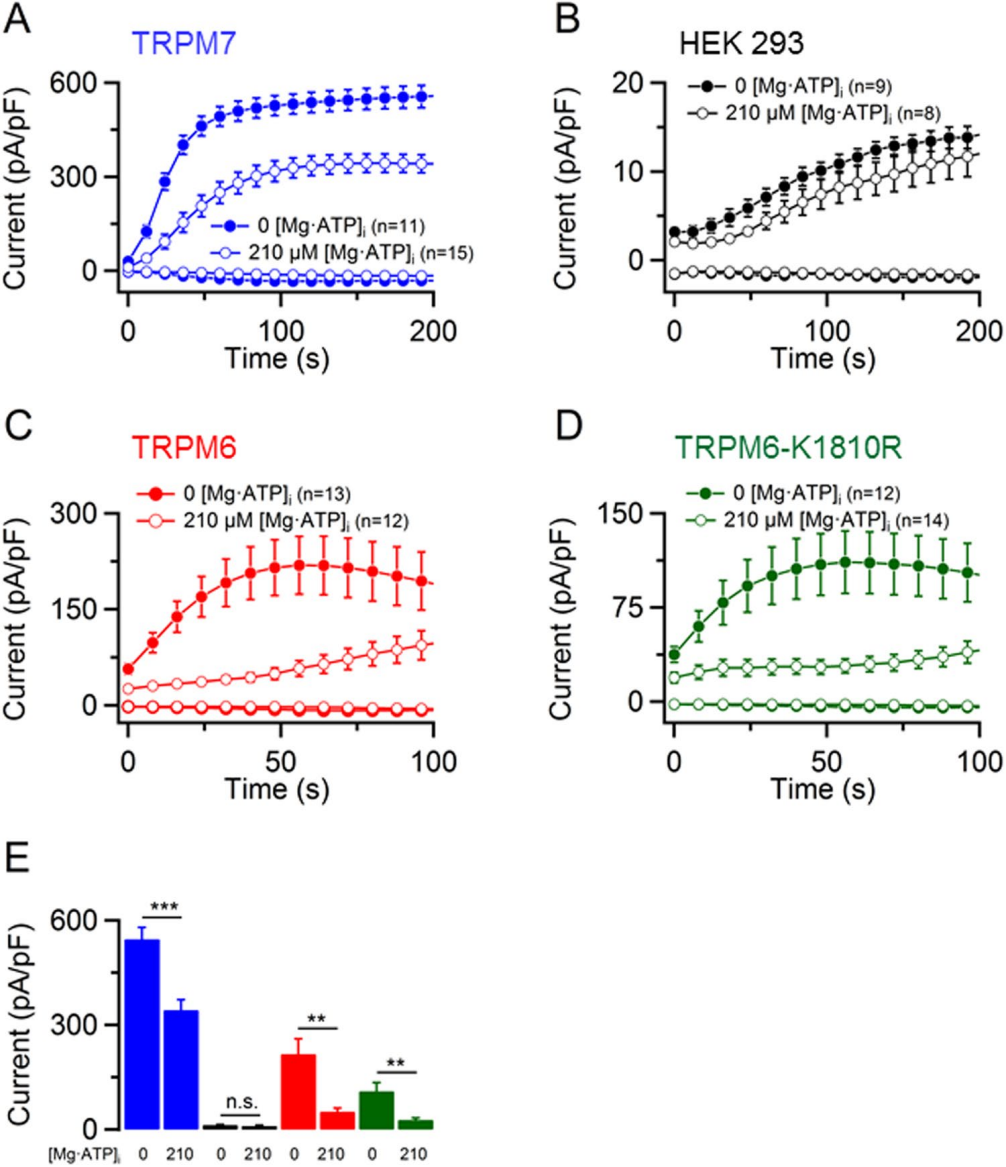
Effects of cytosolic Mg·ATP on mTRPM6 and mTRPM7 currents. (**A**) Whole-cell currents (mean ± SEM) measured in mTRPM7-transfected HEK 293 cells (**A**), untransfected cells (**B**), or cells transfected either by wildtype mTRPM6 (**C**) or mTRPM6-K1810R variant (**D**). Cells were perfused either with the standard [Mg^2+^]_i_-free intracellular solution or with a solution containing 210 µM Mg·ATP (10 µM free Mg^2+^). (**E**) Bar graphs of outward currents (−80 mV) shown in (**A**–**D**). Current amplitudes (mean ± SEM) were extracted at time intervals when the currents were maximally induced by the Mg^2+^-free intracellular solution as follows: at 200 s for mTRPM7 (blue) and untransfected cells (black), 50 s for wildtype mTRPM6 (red) and mTRPM6-K1810R (green). n, number of cells measured; n.s., not significant; **P < 0.01; ***P < 0.001 (two-tailed t-test).

In a physiological saline solution, the apparent K_d_ of Mg·ATP is 50 ± 10 µM^52^ implying that cytosolic Mg·ATP will always co-exist with a certain amount of free Mg^2+^ sufficient to block mTRPM6 regardless of the actual content of Mg·ATP. To test this assumption, we compared the effects of a relatively low concentration of [Mg·ATP]_i_ (210 µM) on mTRPM7 and mTRPM6. We observed that mTRPM7 currents were partially inhibited by Mg·ATP (Fig. 7A,E). Interestingly, endogenous TRPM7-like currents were only modestly affected under these conditions (Fig. 7B,E). As expected, 210 µM [Mg·ATP]_i_ strongly suppressed currents in wild-type mTRPM6 and mTRPM6-K1810R transfected cells (Fig. 7C–E). Hence, mTRPM6 displays an extraordinarily high sensitivity to cytosolic Mg^2+^ independent of the catalytic activity of its kinase domain.

### Functional characterization of heteromeric mTRPM6/7 channel complexes

Our experiments with recombinant mTRPM6 suggest that homomeric mTRPM6 channels, if formed in the cell, will most likely be inactive in the presence of physiological concentrations of cytosolic Mg^2+^ (Fig. 6), which are estimated to be in the range of 0.3–1 mM^53, 54^. Previously, we suggested^38, 39^ that the native TRPM6 protein primarily exists as a subunit of heteromeric channel complexes formed by TRPM6 and TRPM7 (TRPM6/7). Consequently, we asked whether such a paradoxical Mg^2+^ sensitivity of mTRPM6 would look different in mTRPM6/7 complexes. To address this question, we co-transfected mTRPM6 and mTRPM7 expression constructs in HEK 293 cells. Western blot analysis of cell lysates revealed that co-expression of mTRPM6 had no effect on the expression levels of mTRPM7 (Supplementary Fig. S4). Next, we used an immunoprecipitation approach to show that mTRPM6 is able to associate with mTRPM7. mTRPM7 fused to a myc tag (mTRPM7-myc) was expressed in HEK 293 cells either alone or co-expressed with mTRPM7, mTRPM6 and mTRPM5 containing a YFP tag (mTRPM7-YFP, mTRPM6-YFP and mTRPM5-YFP, respectively). mTRPM7-myc was solubilized and immunoprecipitated using an anti-myc antibody. Both cell lysates and immunoprecipitates were subjected to SDS-gel electrophoresis and subsequent immunoblotted with an anti-GFP antibody (cross-reacting with YFP) or anti-myc antibody (Supplementary Fig. S5). As expected, we detected mTRPM7-myc in the cell lysates and immunoprecipitates (Supplementary Fig. S5). In addition, mTRPM7-myc immunoprecipitates contained mTRPM7-YFP and mTRPM6-YFP, but not a more distantly related channel, mTRPM5-YFP (Supplementary Fig. S5). Hence, in line with our previous findings with hTRPM6^38, 39^, mTRPM6 can assemble with mTRPM7 in heteromeric channel complexes.

**Figure 8:**
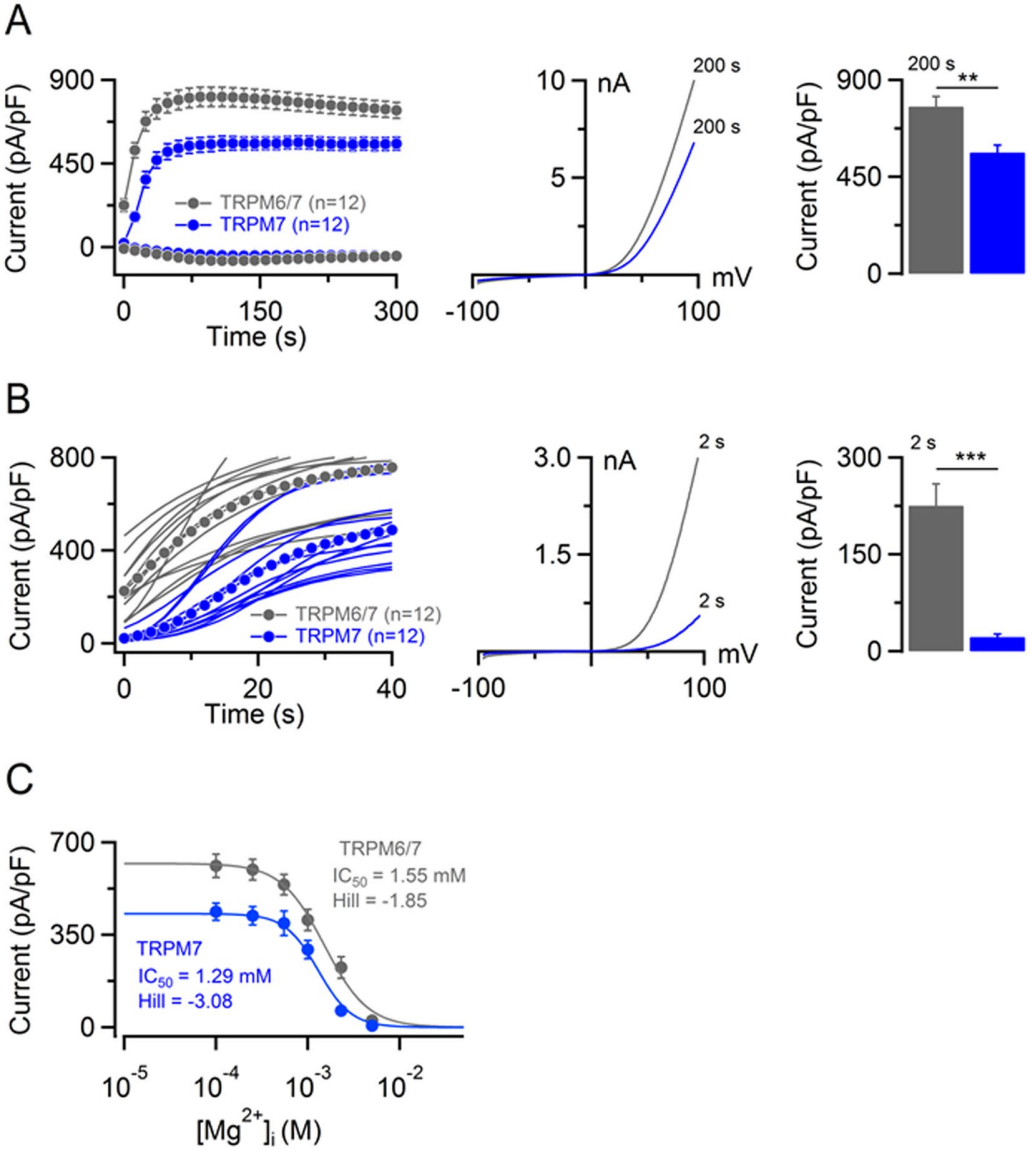
Assessment of mTRPM6/7 currents. (**A**) *Left panel:* Whole-cell currents measured in mTRPM7- (blue) and mTRPM6/7-transfected (grey) HEK 293 cells using the standard [Mg^2+^]_i_-free internal solution and standard external solution. Current amplitudes (mean ± SEM) were acquired at −80 and +80 mV and plotted over time. *Middle panel*: Representative I-V relationships (at 200 s) of currents shown in the *Left panel*. *Right panel:* Bar graphs of outward currents (+80 mV) shown in the *Left panel*. (**B**) A magnification of currents illustrated in (A). *Left panel:* Outward current amplitudes (at +80 mV) acquired from individual cells with the corresponding means (dots). *Middle panel*: Representative I-V relationships of currents (at 2 s) shown in the *Left panel*. *Right panel:* Bar graphs of outward currents (mean ± SEM) at +80 mV in the *Left panel*. n, number of cells measured; **P < 0.01; ***P < 0.001 (two-tailed t-test). (**C**) Dose-dependent inhibition of currents (+80 mV, 200 s) by [Mg^2+^]_i_. Measurements (n = 10–12 cells per concentration) were performed as in (A). n, number of cells measured for individual [Mg^2+^]_i_ tested.

Next, we performed patch-clamp experiments with HEK 293 cells co-transfected with mTRPM6 and mTRPM7 cDNA constructs. In initial experiments, we assessed the currents induced by standard [Mg^2+^]_i_-free internal solution (Fig. 8A). We found that cells co-transfected with 1 µg mTRPM6 and 1 µg mTRPM7 cDNA constructs displayed mTRPM7-like currents substantially larger than cells transfected with 2 µg mTRPM7 cDNA alone (Fig. 8A). In addition, we noted that all cells transfected with mTRPM6/7 cDNAs exhibited large currents already at break-in (Fig. 8B). I-V relationships of these pre-activated currents (Fig. 8B) were indistinguishable from those of fully developed currents (Fig. 8A). In contrast, all solely mTRPM7 expressing cells displayed very small currents after break-in (Fig. 8B). Further analyses revealed that amplitudes of pre-activated currents of mTRPM6/7 were ~10-fold larger than corresponding values obtained with mTRPM7 expression (Fig. 8B) and were comparable with fully activated currents (at 90 s) of mTRPM6 homomers (Fig. 2B). Of note, such constitutive channel activity was not observed in experiments with untransfected HEK 293 cells (Fig. 2B) or in cells transfected by mTRPM6 alone (Fig. 2A). These findings indicate that in resting cells heteromeric mTRPM6/7 channels were active prior to manipulation of intracellular [Mg^2+^]_i_ and [Mg·ATP]_i_ via the patch pipette. Consequently, we asked whether such high constitutive activity of mTRPM6/7 can be attributed to an increased expression of mTRPM6 or mTRPM7 protein in the plasma membranes of cells co-transfected with both constructs as compared to cells expressing only mTRPM6 or mTRPM7. To this end, we isolated the plasma membrane proteins from cells transfected with mTRPM6 or mTRPM7 and from cells co-transfected with mTRPM6 and mTRPM7 cDNA (Supplementary Fig. S6). Western blot analysis revealed that the plasma membrane levels of mTRPM6 and mTRPM7 protein in co-transfected cells were reduced as compared to cells expressing only mTRPM6 or mTRPM7 (Supplementary Fig. S6). Hence, the high channel activity of mTRPM6/7 is unlikely caused by an increased expression of heteromeric channels in the plasma membrane, but represents a qualitative hallmark of mTRPM6/7.

Next, we examined the concentration-dependent suppression of currents by [Mg^2+^]_i_ (Fig. 8C, Supplementary Table S1). Cells expressing mTRPM6/7 exhibited larger current amplitudes over the whole range of [Mg^2+^]_i_ examined (P ≤ 0.0001, F-test). Compared to mTRPM7, we noted only a modest, but statistically significant rightward shift of the concentration-response curve of mTRPM6/7 currents. Thus, the calculated IC_50_ value for mTRPM7 currents was 1.29 mM. Currents in mTRPM6/7 expressing cells were inhibited by [Mg^2+^]_i_ with an IC_50_ value of 1.55 mM (P ≤ 0.001, F-test). These results suggest that mTRPM7 in mTRPM6/7 complexes offsets the exquisite Mg^2+^ sensitivity of mTRPM6 to physiological levels of cytosolic Mg^2+^.

**Figure 9:**
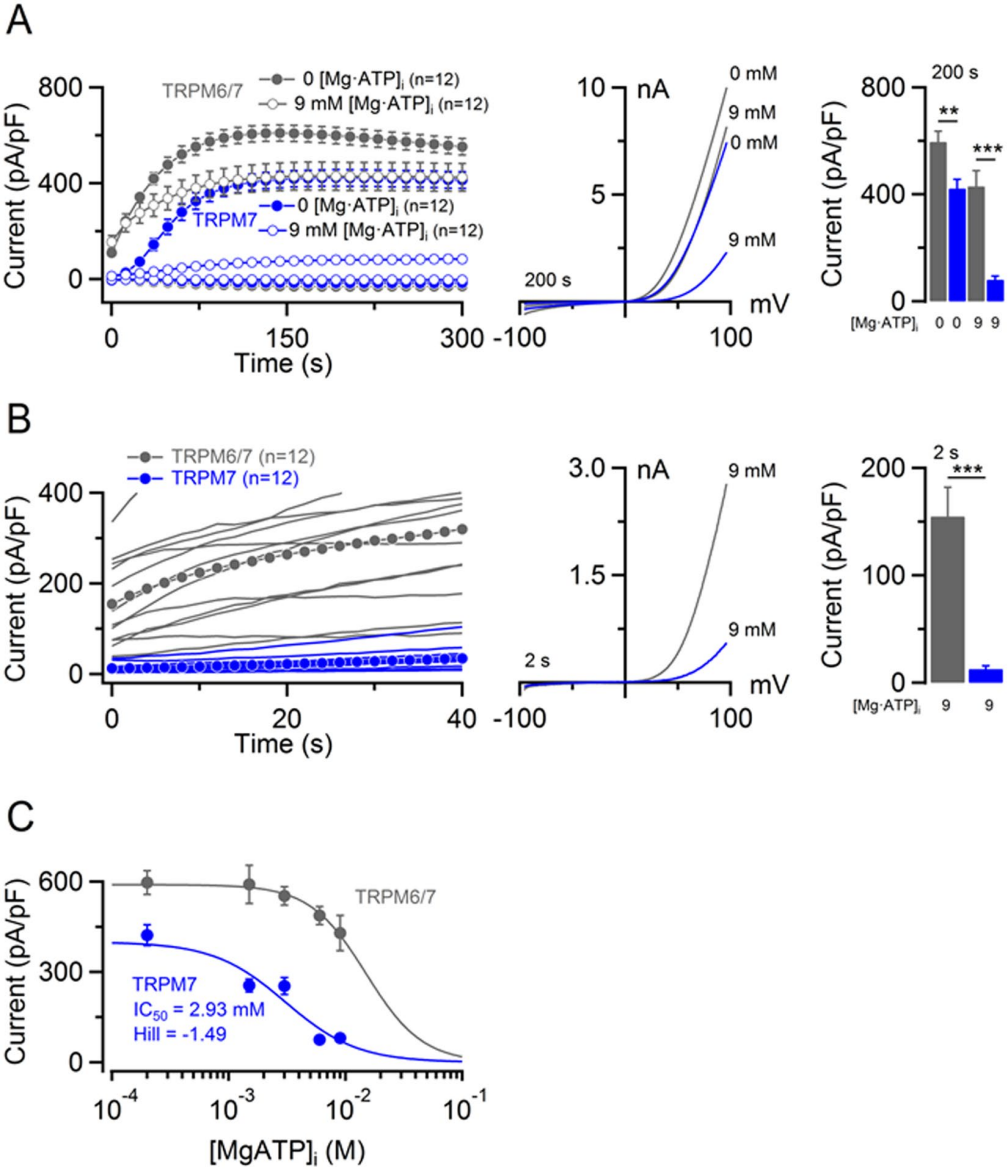
Sensitivity of mTRPM6/7 currents to cytosolic Mg·ATP. (**A**) *Left panel:* Whole-cell currents measured in mTRPM7 (blue) and mTRPM6/7 (grey) transfected HEK 293 cells using internal solutions containing 9 mM [Mg·ATP]_i_ and 250 µM free [Mg^2+^]_i_ (open dots) or only 250 µM free [Mg^2+^]_i_ (closed dots). Current amplitudes (mean ± SEM) were acquired at −80 and +80 mV and plotted over time. *Middle panel*: Representative I-V relationships (at 200 s) of currents shown in the *Left panel*. *Right panel:* Bar graphs of outward currents (−80 mV) shown in the *Left panel*. (**B**) Magnification of currents illustrated in (A). *Left panel:* Outward current amplitudes (at +80 mV) acquired from individual cells with the corresponding means (dots). *Middle panel*: Representative I-V relationships of currents (at 2 s) shown in the *Left panel*. *Right panel:* Bar graphs of outward currents (mean ± SEM) at +80 mV in the *Left panel*. n, number of cells measured; **P < 0.01; ***P < 0.001 (two-tailed t-test). (**C**) Dose-dependent inhibition of mTRPM7 (blue) and mTRPM6/7 (grey) currents (+80 mV, 200 s) by [Mg·ATP]_i_ (250 µM free [Mg^2+^]_i_). Measurements (n = 9–12 cells per concentration) were performed as in (A).

Next, we asked whether mTRPM6/7 would be active in the presence of Mg·ATP. Physiological concentrations of [Mg·ATP]_i_ vary between 2–7 mM in most mammalian cells^53, 54^. First, we studied the effects of relatively high levels of Mg·ATP (Fig. 9A). We found that 9 mM [Mg·ATP]_i_ entailed nearly complete suppression of mTRPM7 channel activity. In contrast, mTRPM6/7 currents were only modestly inhibited by 9 mM [Mg·ATP]_i_ (Fig. 9A). Concordant with experiments shown in Fig. 8B, we observed ~10-fold higher channel activity in mTRPM6/7 expressing cells immediately after break-in (Fig. 9B). Next, we compared the concentration-dependent suppression of mTRPM6/7 and mTRPM7 currents by [Mg·ATP]_i_ (because of experimental limitations, effects of [Mg·ATP]_i_ higher than 10 mM could not be reliably examined). [Mg·ATP]_i_ suppressed mTRPM7 currents with an IC_50_ value of 2.93 mM (Fig. 9C, Supplementary Table S2). In contrast, mTRPM6/7 complexes were characterized by a remarkably low sensitivity to [Mg·ATP]_i_ at all concentrations examined (P ≤ 0.0001, F-test). Such a weak effect of [Mg·ATP]_i_ on mTRPM6/7 currents did not allow for a reliable calculation of an IC_50_ value. Nevertheless, the concentration-response data indicate that more than 15 mM [Mg·ATP]_i_ are required to reduce mTRPM6/7 currents by ~50% (Fig. 9C), suggesting that, in stark contrast to mTRPM7, physiological levels of [Mg·ATP]_i_ will exert only a minor (if any) inhibitory effect on mTRPM6/7 currents.

It has been proposed that the catalytic activity of the hTRPM6 kinase domain regulates the sensitivity of the hTRPM6/7 channels to [Mg·ATP]_i_^27^_._ Therefore, we investigated whether the kinase-inactivating K1810R mutation in mTRPM6 would shift the [Mg·ATP]_i_ sensitivity of the mTRPM6/7 channels into the mTRPM7-like range. We examined cells transiently expressing mTRPM6-K1810R and wildtype mTRPM7 and found that mTRPM6-K1810R behaved similarly to wildtype mTRPM6. Specifically, we found that the standard Mg^2+^-free internal solution induced mTRPM6-K1810R/7 currents (Supplementary Fig. S7) comparable to values displayed by the wildtype mTRPM6/7 channels (Fig. 8A) and that the ‘kinase-dead’ mutation did not offset the low sensitivity of heteromeric channels to 9 mM [Mg·ATP]_i_ (Supplementary Fig. S7). We conclude that the catalytic activity of the mTRPM6 kinase is unlikely to play a major role in this regulatory mechanism.

Finally, we studied whether mTRPM6 and mTRPM6/7 respond differentially to small synthetic molecules acting as activators or inhibitors of the mTRPM7 channel^55^. We first examined the effect of NS8593, a potent mTRPM7 inhibitor^49^, on mTRPM6 currents (Supplementary Fig. S8A). In this experiment, we induced mTRPM6 currents using a Mg^2+^-free pipette solution and externally applied 10 µM NS8593 when mTRPM6 currents were fully developed. We noted that NS8593 caused a rapid irreversible inhibition of mTRPM6 (Supplementary Fig. S8A). Next, we assessed effects of naltriben, a potent agonist of mTRPM7 channel^56^. In these experiments, we used intracellular solutions containing 2 mM [Mg^2+^]_i_ for mTRPM7, whereas mTRPM6 currents were evaluated in the presence of a [Mg^2+^]_i_-free solution or saline containing 1 µM fee [Mg^2+^]_i_. As expected, the external application of 50 µM naltriben led to a fast stimulation of mTRPM7 currents (Supplementary Fig. S8B). Unlike mTRPM7, mTRPM6 did not respond to 50 µM naltriben, neither in the presence of 1 µM [Mg^2+^]_i_ nor in Mg^2+^-free conditions (Supplementary Fig. S8C). It has been reported that 2-aminoethyl diphenylborinate (2-APB) acts as a positive modulator of hTRPM6 and an inhibitor of hTRPM7^27, 43^. In our experimental settings, 200 µM 2-APB reversibly blocked mTRPM7 currents (Supplementary Fig. S9A). In contrast, 200 µM 2-APB potentiated mTRPM6 currents (Supplementary Fig. S9B). Hence, mTRPM6 displays distinct responses to naltriben and 2-APB. Consequently, we asked how these compounds affect mTRPM6/7 currents. We observed that naltriben (50 µM) could modestly potentiate mTRPM6/7 currents, but these changes were not statistically significant (Supplementary Fig. S8D). 200 µM 2-APB triggered a moderate, slow and irreversible rundown of mTRPM6/7 currents (Supplementary Fig. S9C). Taken together, our study offers a set of endogenous and exogenous agents enabling to distinguish mTRPM6, mTRPM7 and mTRPM6/7 currents.

## Discussion

Here we employed a newly cloned mTRPM6 cDNA to provide further evidence in support of the previously suggested concept that native TRPM6 functionally interacts with TRPM7 to maintain transcellular Mg^2+^ transport^37–39^. We show that mTRPM6 and mTRPM7 differentially contribute to regulatory characteristics of heteromeric mTRPM6/7 channel complexes: mTRPM7 is able to offset the very high sensitivity of mTRPM6 to cytosolic Mg^2+^ to physiologically relevant concentrations, whereas mTRPM6 relieves mTRPM7 from inhibition by Mg·ATP. Consequently, in contrast to mTRPM7, the activity of mTRPM6/7 channels will hardly be affected by physiological intracellular concentrations of Mg^2+^ and Mg·ATP. Such a functional fingerprint is most likely not critical for metabolic processes in resting cells, but this mechanism appears to be an indispensable prerequisite for efficient transcellular Mg^2+^ transport in placental and intestinal epithelial cells i.e. when a high and constant uptake of extracellular Mg^2+^ should be uncoupled from the cellular metabolism of Mg^2+^ and Mg·ATP.

TRPM6 is inevitably co-expressed with the ubiquitously present TRPM7 and the mechanism of the non-redundant role of TRPM6 for organismal Mg^2+^ balance is subject to considerable debate. Previously, our group extensively studied recombinant expression of hTRPM6 in HEK 293 cells and in *Xenopus* oocytes^38, 39^. Regardless of the experimental system and plasmid backbone used, we observed that hTRPM6 homomultimers are retained in intracellular membrane compartments. However, co-expression of hTRPM6 and mTRPM7 resulted in co-trafficking of TRPM6/7 channels to the cell surface^38–40^. Current amplitudes of TRPM6/7 complexes were found to be higher than those of TRPM7 homomers^38, 39^. The assembly of recombinant heteromeric TRPM6/7 channel complexes was demonstrated by fluorescence resonance energy transfer (FRET) and co-immunoprecipitation approaches as well as by functional analysis of channel subunits carrying a dominant-negative point mutation in the pore-forming segment^38–40^. More recently, we investigated placental trophoblast stem (TS) cells isolated from gene-modified mice^37^. We observed that a disruption of native mTRPM6 resulted in a reduction of current amplitudes, whereas deletion of mTRPM7 caused complete ablation of endogenous currents. Remarkably, the currents in mTRPM6-deficient TS cells were considerably more sensitive to intracellular Mg·ATP^37^. These findings are consistent with a recent study of Zhang *et al*.^57^ elucidating the contribution of hTRPM6 and hTRPM7 to endogenous currents in human neuroblastoma SHEP-21N cells, and with work of Ryazanova *et al*. examining native currents in mouse embryonic stem (ES) cells expressing mTRPM6 and mTRPM7^5^. The latter reports also showed that genetic ablation of mTRPM7 led to full suppression of TRPM7-like currents. Hence, the functional analysis of native currents entertains the notion that native TRPM6 functions as a subunit of TRPM6/7 complexes where it increases current amplitudes and relieves TRPM7 from inhibition by [Mg·ATP]_i_.

However, it should not go unnoticed that other researchers claimed that hTRPM6 can function independently of TRPM7^42–44^. The latter proposition is based on the finding that transient transfection of pCINeo-hTRPM6-IRES-GFP expression constructs (but not other expression vectors^27^) allows to detect channel activity of hTRPM6 homomultimers. Interestingly, experiments with pCINeo-hTRPM6-IRES-GFP yielded remarkably different results concerning the sensitivity of hTRPM6 to [Mg^2+^]_i_ and [Mg·ATP]_i_. Thus, one group reported that hTRPM6 is suppressed by [Mg^2+^]_i_ and [Mg·ATP]_i_ with IC_50_ values of 510 µM and 1.3 mM, respectively^42, 44^. In contrast, another laboratory observed that pCINeo-hTRPM6-IRES-GFP evoked currents that were completely insensitive to 3–9 mM [Mg·ATP]_i_, whereas [Mg^2+^]_i_ efficiently blocked hTRPM6 currents with a physiologically irrelevant IC_50_ of 29 µM^27^. The reasons for such discrepancies still remain unclear. In particular, there is no sensible argument as to why only one specific expression plasmid should allow for functional expression of hTRPM6.

Since the functional characteristics of heterologously expressed hTRPM6 are surrounded by considerable controversy, we focussed on a newly isolated murine TRPM6 clone. We noted that recombinant mTRPM6 homomultimers could be functionally expressed irrespective of the vector backbone used. Although such an overexpression system does not fully recapitulate the properties of native mTRPM6 (e.g. endogenous mTRPM6 at rather low protein levels is active only in the presence of mTRPM7^37^), this *in vitro* model may be instrumental in dissecting functional hallmarks of the kinase and channel activity of mTRPM6 upon co-expression with recombinant mTRPM7. We noted that overexpression of mTRPM6 in HEK 293 cells yielded currents significantly (~3-fold) smaller than mTRPM7 currents. In contrast to mTRPM7, mTRPM6 currents rapidly inactivated, especially when the mTRPM6 expressing cells were exposed to monovalent cation-based external solutions. We also noted that the mTRPM6 channel is more selective for Zn^2+^ as compared to Mg^2+^, Ca^2+^ and Ba^2+^, resembling the ion permeation profile of mTRPM7. Surprisingly, the mTRPM6 channel was found to be highly sensitive to cytosolic Mg^2+^: mTRPM6 was inactive even in the presence of a nominally Mg^2+^-free intracellular solution. The catalytic activity of the mTRPM6 kinase does not appear to play a role in the channel’s high sensitivity to [Mg^2+^]_i_. In this context, our findings are in accord with results of Zhang *et al*. showing supressed hTRPM6 currents in the presence of non-physiologically low concentrations of [Mg^2+^]_i_ (IC_50_ of 29 µM)^27^. Therefore, mTRPM6 as well as hTRPM6 homomers, if formed, will be inactive in the presence of physiological levels of cytosolic Mg^2+^ or [Mg·ATP]_i_.

Finally, we examined how mTRPM6 may modulate the response of mTRPM7 to [Mg^2+^]_i_ and [Mg·ATP]_i_. We noted that cells co-transfected with mTRPM6 and mTRPM7 displayed currents amplitudes significantly larger than cells only harbouring recombinant mTRPM7, mimicking the situation in TS cells^37^. Of note, mTRPM6/7 channels displayed high channel activity immediately after break-in of the patch, suggesting that mTRPM6/7 complexes are constitutively active in the presence of steady-state cytosolic concentrations of Mg^2+^ and Mg·ATP. In line with this concept, we observed that, contrary to mTRPM7, mTRPM6/7 currents were only slightly inhibited by physiological levels of [Mg·ATP]_i_ (3–9 mM) and were significantly more active in the presence of physiological [Mg^2+^]_i_ levels (0.5–1 mM). These findings are concordant with the study of Zhang *et al*. reporting that hTRPM6/7 heteromers are not sensitive to 9 mM [Mg·ATP]_i_^27^ and additionally with our assessment of native currents in TRPM6- *vs* TRPM7-deficient TS cells^37^. Moreover, we found that the catalytic activity of the mTRPM6 kinase does not contribute to the low sensitivity of the mTRPM6/7 channels to [Mg·ATP]_i_. Lastly, mTRPM6/7 displayed differential responses to small organic compounds such as naltriben and 2-APB as compared to mTRPM6 or mTRPM7.

To summarize, our findings indicate that mTRPM6 and mTRPM7 contribute differentially to key functional characteristics of mTRPM6/7 complexes thereby functionally defining a new type of channel that remains active in the presence of physiological concentrations of [Mg^2+^]_i_ and [Mg·ATP]_i_. Consequently, mTRPM6/7 channels will be able to maintain a constant supply of Mg^2+^ for the organism regardless of the actual metabolic state of epithelial cells. In contrast, the channel activity of mTRPM7 homomers is tightly controlled by cytosolic levels of [Mg^2+^]_i_ and [Mg·ATP]_i_ and, thus, closely linked to cellular metabolism. Such a mechanistic model provides a plausible answer for the hitherto open question as to why in transporting epithelia TRPM6 function cannot be replaced by other ion channels including TRPM7.

## Methods

### Molecular biology, *in silico* analysis and cell culture

In the course of NCBI GenBank data mining, a predicted mRNA sequence of *Mus musculus Trpm6* gene was identified (NM_153417.1) and was therefore used for cloning of a full-length *Trpm6* cDNA. TRIzol reagent (Thermo Fisher Scientific) was used for extraction of total RNA from the whole lung of C57BL/J mice and SuperScript II reverse transcriptase (Thermo Fisher Scientific) for first strand synthesis. The Expand High Fidelity polymerase enzyme system (Roche) was used for PCR amplification of different overlapping segments of the predicted TRPM6 ORF. We used the following primer pairs: mM6for1 5′-GAGAATGCAGGTCAAGAAGCAATC-3′ and mM6rev3 5′-TGCCCACAGTCCCATCAT-3′ with PCR settings: 94 °C 3′, 94 °C 30″, 55 °C 30″, 72 °C 1′, 35 cycles, 72 °C 5′ (PCR product 752 bp encompassing exons 1–7 of *Trpm6*); mM6for2 5′-TGCCCTAAAAGCCCATTCCTCTAA-3′ and mM6rev4 5′-CGTCCCCCTCTTCCTGGTCCTGT-3′ with PCR settings: 94 °C 3′, 94 °C 30″, 58 °C 30″, 72 °C 3′, 35 cycles, 72 °C 5′ (PCR product 2873 bp corresponding to exons 2–24 of *Trpm6*); mM6for3 5′-ATGGCGCCTGGCTCGTGACA-3′ and mM6rev2 5′-ACCACCGTCTTCCTTCATCATCTTTTT-3′ with PCR settings: 94 °C 3′, 94 °C 30″, 58 °C 30″, 72 °C 4′, 35 cycles, 72 °C 5′ (PCR product 3077 bp comprising sequence of exons 22–39 of *Trpm6*). The obtained PCR products were inserted into pcDNA3.1 vector by a TOPO-cloning approach (pcDNA3.1/V5-His TA-TOPO kit, Thermo Fisher Scientific) and confirmed by sequencing (Eurofins Genomics, Ebersberg, Germany). Next, the full-length *Trpm6* cDNA (NCBI accession KX375810) was generated by in-frame subcloning of the latter cDNA fragments either in pcDNA3.1 or in pIRES2-EGFP (Clontech) expression vectors using standard molecular biological techniques. Translation *in silico* (DNASTAR Lasergene software) of the cloned TRPM6 cDNA produced a 2028-aa ORF that matched to the predicted ORF sequence in NM_153417.1. 3D model of the TRPM6 kinase domain structure were generated as reported previously^29, 39^ using MODELLER (modbase.compbio.ucsf.edu/modweb) and UCSF Chimera (www.cgl.ucsf.edu/chimera).

K1810R and T1730A point mutations in mTRPM6 cDNA (both in pIRES2-EGFP vector) were introduced by site-directed mutagenesis (QuikChange, Stratagene). To generate mTRPM6 with a C-terminal myc tag in the pcDNA3.1/V5-His TA-TOPO vector (mTRPM6-myc) and with a C-terminal yellow fluorescent protein (YFP) tag in pcDNA3.1/V5-His TA-TOPO vector (mTRPM6-YFP), a STOP codon in the mTRPM6 cDNA was replaced by a *Sal*I restriction site through site-directed mutagenesis followed by in-frame sub-cloning of myc or YFP coding sequence as reported previously^26, 43^. All generated mTRPM6 cDNA variants were verified by sequencing.

mTRPM7 (in pIRES2-EGFP vector), mTRPM7-myc (in pcDNA3.1/V5-His TA-TOPO vector) and mTRPM7-myc (in pcDNA3.1/V5-His TA-TOPO) were reported previously^29, 38, 58^. mTRPM5-YFP (in pcDNA3.1/V5-His TA-TOPO) was described earlier^59, 60^. Likewise, the hTRPM6 expression construct (pCINeo-hTRPM6-IRES-GFP) has been reported before^27, 42^.

Human embryonic kidney (HEK) 293 cells were grown at 37 °C and 5% CO_2_ in Eagle’s minimum essential medium (MEM, Sigma Aldrich) supplemented with 10% fetal bovine serum (FBS, Thermo Fisher Scientific), 100 U/ml penicillin and 100 µg/ml streptomycin (P/S, Sigma-Aldrich). Cells were transiently transfected by 2 µg expression constructs using Lipofectamine 2000 reagent (Thermo Fisher Scientific). In some experiments, 1 µg of mTRPM6 and 1 µg mTRPM7 cDNA constructs were co-transfected in HEK 293 cells. Patch-clamp experiments were performed 20–24 h after transfection. Successfully transfected cells were identified by their green fluorescence when illuminated at 480 nm.

The HEK-293 T-REx cell line stably expressing hTRPM6 was cultured as reported previously^27, 50^. hTRPM6 overexpression was induced by adding 1 µg/ml doxycycline (Thermo Fisher Scientific) to the growth medium. Patch-clamp experiments were performed 20–24 h after induction.

### Transient expression of mTRPM6 in *Trpm7*-deficient trophoblasts stem (TS) cells

*Trpm7*-gene deficient TS cells were generated as described before^37^. TS cells were incubated in a humidified cell culture incubator (Heraeus, Thermo Fisher Scientific) at 37 °C and 5% CO_2_ in RPMI 1640 medium (Thermo Fisher Scientific) supplemented with 20% fetal bovine serum (ES type, Thermo Fisher Scientific), 1 mM sodium pyruvate (cell culture type, Sigma-Aldrich), 100 µM β-mercaptoethanol (Sigma-Aldrich), 50 µg/ml streptomycin and 50 U/ml penicillin (Thermo Fisher Scientific), 1.0 µg/ml heparin (cell culture type, Sigma-Aldrich), 25 ng/ml human recombinant FGF4 (R&D systems), 5 ng/ml human recombinant TGF-β1 (R&D systems), 10 ng/ml recombinant activin A (R&D systems) and an additional 10 mM MgCl_2_. For transient expression of mTRPM6, 1 × 10^5^ TS cells were electroporated with 2 µg cDNA by applying two voltage pulses (1000 V, 30 ms) using Neon Transfection System (Thermo Fisher Scientific). The TS cells were studied 24 h after electroporation.

### Generation of (p)T1730 mTRPM6-specific antibody and Western blot analysis

To generate a polyclonal (p)T1730 mTRPM6-specific antibody, rabbits were immunized with a phosphorylated peptide AcNH-RLSQ(p)TIPFTPIQC-CONH_2_ coupled via its C-terminal cysteine residue to keyhole limpet hemacyanin (Eurogentec, Belgium). The generated serum was subjected to two rounds of peptide affinity chromatography. First, a fraction of antibody was purified using the phosphorylated peptide. Second, the isolated antibody was followed by an additional round of chromatography using a non-phosphorylated variant of the peptide (AcNH-RLSQTIPFTPIQC-CONH_2_) in order to deplete a fraction of antibody with cross-reactivity to a non-phosphorylated mTRPM6 protein. The final fraction of anti-(p)T1730 mTRPM6 antibody was aliquoted and stored at −80 °C.

To assess the mTRPM6 kinase activity, the anti-(p)T1730 mTRPM6 antibody was used to probe cell lysates obtained from HEK 293 transiently transfected with mTRPM6 cDNA variants. In some experiments (Fig. 1D) transfected cells were cultured for 12 h in the presence of TG100–115 (Absource Diagnostic GmbH). The lysis buffer (Pierce IP Lysis Buffer, Pierce) contained protease inhibitor and phosphatase inhibitor cocktails (Biotool). Aliquots of the cell lysates were mixed (1:1) with 2x Laemmli buffer, heated at 70 °C for 10 min and cooled on ice. The samples were separated by SDS-PAGE (6% acrylamide/bis-acrylamide, Carl Roth) and electroblotted on nitrocellulose membranes (GE Healthcare Life Science). After blocking with 5% (w/v) non-fat dry milk in Tris-buffered saline with 0.1% Tween 20 (TBST), the membranes were probed by (p)T1730 mTRPM6-specific antibody (1 µg/ml in TBST with 5% (w/v) BSA), followed by washing in TBST, incubation with a horseradish peroxidase-coupled anti-rabbit lgG (Cell Signaling Technology; 1:1000 in TBST with 5% (w/v) non-fat dry milk) and washing again in TBST. Blots were exposed in a luminescence imager (Peqlab/VWR, Germany). Expression levels of mTRPM6 were examined using a guinea pig anti-mTRPM6 polyclonal antibody (ab47017, Abcam; 1:4000) and a horseradish peroxidase-coupled anti-guinea pig lgG (Acris; 1:1000). Western blot assessment of mTRPM7 was performed analogously to mTRPM6 using a rabbit anti-TRPM7 monoclonal antibody (EPR4582, Abcam; 1:2000).

To assess expression levels of mTRPM6 and mTRPM7 in the plasma membranes, HEK 293 cells cultured in 100-mm cell culture dishes (~70% confluency) were transiently transfected with 10 and 20 µg mTRPM6, 10 and 20 µg mTRPM7 or co-transfected with 10 µg mTRPM6 together with 10 µg mTRPM7 (all in pIRES2-EGFP vector). 24 h after transfection, cells were collected by centrifugation and the cell pellets were washed twice with ice-cold PBS. Plasma membrane proteins were extracted using the Minute^TM^ plasma membrane protein isolation kit (Invent Biotechnologies) according to the manufacturer’s protocol. The obtained samples of plasma membrane proteins (50 μl) were mixed with 50 μl 2x Laemmli buffer, heated at 70 °C for 10 min and cooled on ice. Next, Western blot analysis of mTRPM6 and mTRPM7 (1/3 of the plasma membrane samples) was performed as described above. The plasma membranes marker Na^+^/K^+^ ATPase was probed using an anti-Na^+^/K^+^ ATPase antibody (EP18459-HRP, Abcam; 1:5000).

### Co-immunoprecipitation of mTRPM7 and mTRPM6

Experiments were performed as reported previously^61^ with several modifications. HEK 293 cells maintained in 100-mm cell culture dishes (~70% confluency) were transiently transfected with 10 µg mTRPM7-myc, 10 µg mTRPM6-YFP and 10 µg mTRPM5-YFP or co-transfected with 10 µg mTRPM7-myc together with 10 µg mTRPM7-YFP, mTRPM6-YFP and mTRPM5-YFP expression constructs. 24 h after transfection, cells were lysed in 1 ml of ice-cold lysis buffer (1 ml of PBS supplemented with 1% Triton X-100 and a protease inhibitor cocktail). Cell lysates were centrifuged at 4 °C (10 min at 10,000 × *g*; 1 h at 30,000 × *g*) and 900 μl of the supernatants were further mixed with an anti-myc 9E10 antibody (M5546, Sigma-Aldrich) immobilized on Protein A/G magnetic beads (88802, Thermo Fisher Scientific). For immobilization, 2 μl anti-myc antibody was incubated with 20 μl (0.2 mg) Protein A/G magnetic beads in 1 ml of the lysis buffer overnight at 4 °C. Afterwards, magnetic beads were washed three times in 1 ml lysis buffer at 4 °C, collected using a magnetic stand, passed to the cell lysates (900 μl) and incubated overnight at 4 °C. Next, beads were washed three times in 1 ml lysis buffer, collected using a magnetic stand, re-suspended in 100 μl 2x Laemmli buffer, heated at 70 °C for 10 min and cooled on ice. 1/5 of the immunoprecipitate samples and aliquots of the cell lysates (10 μl mixed with 10 μl 2x Laemmli buffer) were subjected to Western blot analysis as described above using an anti-GFP antibody (ab290, Abcam; 1:1000 in TBST with 5% (w/v) BSA) or anti-myc antibody (9B11, Cell Signaling Technology; 1:1000 in TBST with 5% (w/v) BSA).

### Electrophysiological techniques

Patch clamp experiments were performed as reported previously^49, 62, 63^ with a few modifications. Whole-cell currents were measured using an EPC10 patch-clamp amplifier and PatchMaster software (Harvard Bioscience). Voltages were corrected for a liquid junction potential of 10 mV. Currents were elicited by a ramp protocol from −100 mV to +100 mV over 50 ms acquired at 0.5 Hz and a holding potential of 0 mV. Inward and outward current amplitudes were extracted at −80 mV and +80 mV and were normalized to cell size as pA/pF. Capacitance was measured using the automated capacitance cancellation function of EPC10. Patch pipettes were made of borosilicate glass (Science Products) and had resistance 2–3.5 MΩ.

Unless stated otherwise, a standard extracellular solution contained (in mM): 140 NaCl, 2.8 KCl, 1 CaCl_2_, 2 MgCl_2_, 10 HEPES-NaOH, and 11 glucose (all from Sigma-Aldrich), pH 7.2. Effects of NS8593 (Tocris), 2-aminoethyl diphenylborinate (2-APB; Sigma-Aldrich) and naltriben (Tocris) were examined by adding the compounds to the standard extracellular solution. A divalent cation-free (DVF) extracellular solution contained (in mM) 140 NaCl, 2.8 KCl, 11 glucose, 5 Na-EDTA and 10 HEPES-NaOH, pH 7.2. In assessment of ion permeation profiles of mTRPM6 and mTRPM7, the extracellular solutions contained: 10 mM HEPES-NaOH, 260 mM mannitole and 10 mM of individual divalent cations (ZnCl_2_, MgCl_2_, CaCl_2_ or BaCl_2_), and were adjusted to pH 7.0 (to prevent the precipitation of ZnCl_2_). Solutions were adjusted to 290 mOsm using a Vapro 5520 osmometer (Wescor Inc).

The standard Mg^2+^-free intracellular ([Mg^2+^]_i_) pipette solution contained (in mM): 120 Cs-glutamate, 8 NaCl, 10 Cs-EGTA, 5 Cs-EDTA, 10 HEPES-CsOH, pH 7.2. In some measurements, we varied free [Mg^2+^]_i_ levels as follows. A nominally [Mg^2+^]_i_-free intracellular solution contained (in mM) 140 Cs-glutamate, 8 NaCl, 10 HEPES-CsOH, pH 7.2. A pipette solution with 1 µM free [Mg^2+^]_i_ comprised (in mM): 140 Cs-glutamate, 8 NaCl, 0.0016 MgCl_2_, 10 Cs-EGTA, 10 HEPES-CsOH, pH 7.2. An intracellular solution with 2 mM free [Mg^2+^]_i_ contained (in mM): 120 Cs-glutamate, 8 NaCl, 2 Cs-EDTA, 10 HEPES-CsOH, 4 MgCl_2_, pH 7.2. A pipette solution with 210 µM Mg·ATP and 10 µM free [Mg^2+^]_i_ contained (in mM): 140 Cs-glutamate, 8 NaCl, 3 Cs-EDTA, 10 HEPES-CsOH, 2.5 Mg·ATP (Sigma-Aldrich), pH 7.2. Concentrations of [Mg·ATP]_i_ and free [Mg^2+^]_i_ were calculated using WebMaxC (maxchelator.stanford.edu).

Data are presented as means ± standard error of the mean (SEM). Data showed normal distribution. Unless indicated differently, data were compared by a two-tailed t-test. For multiple comparisons in Fig. 2D, Supplementary Fig. S7, and Supplementary Fig. S9, ANOVA (GraphPad Prism 6.0 software) was used. Significance was accepted at P ≤ 0.05.

To determine [Mg^2+^]_i_ and [Mg·ATP]_i_ dose responses, the intracellular pipette solutions were prepared as outlined in Supplementary Table S1 and Supplementary Table S2, respectively. To determine IC_50_ values for inhibitory effects of [Mg·ATP]_i_ and [Mg^2+^]_i_ on mTRPM6 and mTRPM7 currents, data were fitted with the following equation:

$${\rm{E}}({\rm{c}})={{\rm{E}}}_{{\rm{\min }}}+({{\rm{E}}}_{{\rm{\max }}}-{{\rm{E}}}_{{\rm{\min }}})\times (1/(1+{({{\rm{IC}}}_{50}/{\rm{c}})}^{{\rm{h}}}))$$

with E being the effect/current at a given concentration c of inhibitor, E_min_ the minimal effect/current, E_max_ the maximally achievable effect, IC_50_ the half-maximal concentration and h the Hill factor. Statistical analysis of dose-response curves and IC_50_ values (Fig. 8C and Fig. 9C) was performed using the extra sum-of-squares F test with the threshold P ≤ 0.05 (GraphPad Prism 6.0).

## Electronic supplementary material

Supplementary information and figures
