# Supplementary material for: TRPM6 and TRPM7 differentially contribute to the relief of heteromeric TRPM6/7 channels from inhibition by cytosolic Mg2+ and Mg·ATP
Source: Sci Rep. 2017 Aug 18;7:8806. doi: 10.1038/s41598-017-08144-1 (PMC5562840; doi:10.1038/s41598-017-08144-1)

Supplementary information

TRPM6 and TRPM7 differentially contribute to the relief of heteromeric TRPM6/7 channels from inhibition by cytosolic Mg^{2+} and Mg·ATP

Authors:

Silvia Ferioli[1], Susanna Zierler[1], Joanna Zaißerer[1], Johann Schredelseker[1], Thomas Gudermann[1,2,3]* and Vladimir Chubanov[1]*

*__Contact:__

vladimir.chubanov@lrz.uni-muenchen.de; thomas.gudermann@lrz.uni-muenchen.de

Affiliations:

[1]Walther Straub Institute of Pharmacology and Toxicology, LMU Munich, Munich, Germany; [2]German Center for Lung Research, Munich, Germany; [3]German Centre for Cardiovascular Research, Munich Heart Alliance, Munich, Germany

Supplementary Figure legends

Supplementary Figure S1. Assessment of hTRPM6 in HEK 293 cells.

(**A**) *Left panel:* Whole-cell currents measured in hTRPM6- (pCINeo-hTRPM6-IRES-GFP) transfected HEK 293 cells. To induce hTRPM6 currents, cells were perfused either with a standard $[Mg^{2+}]_i$-free intracellular solution or with a solution containing 1 µM free $[Mg^{2+}]_i$. Current amplitudes (mean ± SEM) were acquired at -80 and +80 mV and plotted over time. *Right panel:* Representative current-voltage (I-V) relationships of currents at 200 s illustrated in *Left panel*. (**B**) Tet-induced (1 µg/ml doxycycline, 20-24 h) and uninduced HEK-293 T-Rex cells stably expressing hTRPM6 were examined as in (A) using the standard $[Mg^{2+}]_i$-free intracellular solution. Current amplitudes (mean ± SEM) were acquired at -80 mV and plotted over time. For comparison, mTRPM6 currents measured in HEK 293 cells transiently transfected with pIRES2-mTRPM6-EGFP (already shown in Fig. 2A) and hTRPM6 currents in HEK 293 cells transiently transfected with pCINeo-hTRPM6-IRES-GFP (already shown in (A)) are also included. n, number of cells measured.

Supplementary Figure S2. Functional expression of mTRPM6 in HEK 293 cells and trophoblast stem (TS) cells.

(**A**) *Left panel:* mTRPM6 in pcDNA3.1 was co-transfected with an EGFP construct (pcDNA3.1) in HEK 293 cells and whole-cell currents were measured at -80 and +80 mV in EGFP-positive cells similarly as described in Fig. 2A using the standard $[Mg^{2+}]_i$-free intracellular solution. Current amplitudes (mean ± SEM) were acquired at -80 and +80 mV and plotted over time. *Right panel:* Representative I-V relationship obtained at 70 s for currents shown in the *Left panel*. (**B**) Comparison of whole-cell currents measured in *Trpm7*-deficient TS cells electroporated with mTRPM6 (pIRES2-mTRPM6-EGFP). TS cells were perfused either with a standard $[Mg^{2+}]_i$-free intracellular solution or with a solution containing 1 µM free $[Mg^{2+}]_i$. Current amplitudes (mean ± SEM) were acquired at -80 and +80 mV and plotted over time. Note that mTRPM6 currents measured in the absence of $[Mg^{2+}]_i$ were already shown in Fig. 2E. n, number of cells measured.

Supplementary Figure S3. Examining of Ba^{2+} permeability of mTRPM7 and mTRPM6.

(**A**) *Left panel:* Whole-cell currents were recorded in mTRPM7-transfected HEK 293 cells using the standard $[Mg^{2+}]_i$-free internal solution and standard external solution. When currents started to develop, cells were subsequently exposed to the external solution containing 10 mM Ba^{2+} as indicated by a bar. Data are shown as I/Imax ± SEM (Imax value was obtained in a ramp before application of 10 mM Ba^{2+}). *Middle panel*: Representative I-V relationships of inward currents obtained before (blue) and during (red) application of 10 mM Ba^{2+} as indicated in the *Left panel*. *Right panel*: Bar graphs of inward currents (-80 mV, mean I/Imax ± SEM) obtained before (blue) and during (red) application of 10 mM Ba^{2+} as indicated in the *Left panel*. (**B**) Changes in the mTRPM6 currents by exposure of cells to the external solution containing 10 mM Ba^{2+}. Measurements and analysis were performed similarly to (A). n, number of cells measured; * $P < 0.05$; *** $P < 0.001$ (two-tailed t-test).

Supplementary Figure S4. Western blot analysis of HEK 293 cells expressing mTRPM6, mTRPM7 and mTRPM6/7.

HEK 293 cells were transiently transfected by mTRPM6 or/and mTRPM7 cDNA constructs and cell lysates were probed either with an anti-mTRPM6 antibody (α-TRPM6, *Upper* panel) or an anti-mTRPM7 antibody (α-TRPM7, *Lower* panel). The expected locations of proteins are indicated by red arrows. Representative blots are shown. The experiment was repeated three times with similar results.

Supplementary Figure S5. Co-immunoprecipitation of mTRPM6 and mTRPM7.

mTRPM7 was C-terminally tagged with a myc tag (M7-myc) and expressed alone or co-expressed with YFP-tagged mTRPM7 (M7-YFP), mTRPM6 (M6-YFP) and mTRPM5 (M5-YFP) in HEK 293 cells as indicated above the panels. Protein A/G magnetic beads and an anti-myc antibody were used to immunoprecipitate channel complexes from the corresponding cell lysates. Immunoprecipitates (IP) and cell lysates (Input) were analysed by immunoblotting with an anti-GFP antibody (α-GFP) cross-reacting with YFP tags or anti-myc antibody (α-myc). The

expected location of the tagged TRPM proteins are indicated on the right side of the blots. Representative blots are shown. The experiment was repeated two times with similar results.

Supplementary Figure S6. Western blot analysis of the plasma membranes from HEK 293 cells expressing mTRPM6, mTRPM7 and mTRPM6/7.

HEK 293 cells were transiently transfected by mTRPM6 or/and mTRPM7 cDNA constructs and plasma membranes were probed either by an anti-TRPM6 antibody, anti-TRPM7 antibody (α-TRPM7) and an antibody directed against the plasma membranes marker Na^+/K^+ ATPase (α-Na^+/K^+ ATPase). The expected locations of the proteins are indicated by red arrows. Representative blots are shown. The experiment was repeated three times with similar results.

Supplementary Figure S7. Co-expression of mTRPM6-K1810R and mTRPM7 in HEK 293 cells.

(**A**) Whole-cell currents measured in HEK 293 cells co-expressing mTRPM6-K1810R and wildtype mTRPM7 using the standard $[Mg^{2+}]_i$-free internal solution and solutions containing either 250 µM free $[Mg^{2+}]_i$ or 9 mM $[Mg{\cdot}ATP]_i$ and 250 µM free $[Mg^{2+}]_i$. (**B**) Bar graphs of outward currents at 200 s (+80 mV, mean ± SEM) shown in (A). n, number of cells measured; n.s., not significant; ** $P < 0.01$ (ANOVA).

Supplementary Figure S8. Effect of NS8593 and naltriben on mTRPM6 and mTRPM6/7 currents.

(**A**) Inhibition of mTRPM6 currents by 10 µM NS8593. *Left panel*: Whole-cell currents measured in mTRPM6-transfected HEK 293 cells. Current amplitudes (mean ± SEM) measured at -80 and +80 mV were plotted over time. Currents were induced using the standard $[Mg^{2+}]_i$-free intracellular solution and the standard external solution. When currents started to develop, cells were exposed to 10 µM of NS8593 as indicated by the black bar. A subset of mTRPM6-positive cells was examined without application of NS8593. *Middle panel*: Representative I-V relationships obtained from individual ramps before (blue) and during (red

and green) application of NS8593 as indicated in the *Left panel*. *Right panel*: Bar graphs of outward currents (+80 mV, mean ± SEM) obtained from untreated (green) and NS8593-treated cells (red) as indicated in the *Left and Middle panels*. *** P < 0.001 (two-tailed t-test). (**B**) Stimulation of mTRPM7 currents by naltriben. Current amplitudes (mean ± SEM) were measured at -80 and +80 mV and plotted over time. Currents were induced using an intracellular solution containing 2 mM free $[Mg^{2+}]_i$ and the standard external solution. Cells were exposed to 50 µM naltriben as indicated by the black bar. (**C**) Assessment of 50 µM naltriben effects on mTRPM6 currents. Measurements were performed and analysed similarly to (B) using either the standard $[Mg^{2+}]_i$-free intracellular solution (closed symbols) or solution containing 1 µM free $[Mg^{2+}]_i$ (open symbols). n.s., not significant (two-tailed t-test). (**D**) Assessment of 50 µM naltriben effects on mTRPM6/7 currents. Measurements were performed and analysed similarly to (B). n.s., not significant (ANOVA); n, number of cells measured.

Supplementary Figure S9. Effect of 2-aminoethyl diphenylborinate (2-APB) on mTRPM7, mTRPM6 and mTRPM6/7 currents.

(**A**) Inhibition of mTRPM7 currents by 2-APB. *Left panel*: Whole-cell currents measured in mTRPM7-transfected HEK 293 cells. Current amplitudes (mean ± SEM) measured at -80 and +80 mV were plotted over time. Currents were induced using the standard $[Mg^{2+}]_i$-free intracellular solution and the standard external solution. When currents started to develop, the cells were exposed to 200 µM of 2-APB as indicated by the black bar. *Middle panel*: Representative I-V relationships obtained from individual ramps before (blue) and during (red) application of 2-APB as indicated in the *Left panel*. *Right panel*: Bar graphs of outward currents (+80 mV, mean ± SEM) obtained from untreated (blue) and 2-APB-treated cells (red) as indicated in the *Left and Middle panels*. *** P < 0.001 (two-tailed t-test). (**B**) Potentiation of mTRPM6 currents by 200 µM 2-APB. Experiments were performed and analysed similarly to (A). * P < 0.05; ** P < 0.01 (two-tailed t-test). (**C**) Effects of 200 µM 2-APB on mTRPM6/7 currents. Experiments were conducted as in explained in (A). *** P < 0.001 (ANOVA). n, number of cells measured.

Supplementary Table S1. Composition of the intracellular pipette solutions used for determination of free $[Mg^{2+}]_i$ dose-responses.

[a]$[Mg^{2+}]_i$ (mM)	Cs-glutamate (mM)	NaCl (mM)	Cs-HEPES (mM)	Cs-EGTA (mM)	$MgCl_2$ (mM)
0.10	140	8	10	10	0.16
0.25	140	8	10	10	0.38
0.55	140	8	10	10	0.83
1.00	140	8	10	10	1.50
2.30	120	8	10	10	3.40
5.00	120	8	10	10	7.10

[a]$[Mg^{2+}]_i$ was calculated using WebMaxC software.

Supplementary Table S2. Composition of the intracellular pipette solutions used for determination of $[Mg \cdot ATP]_i$ dose-responses.

[a]$[Mg \cdot ATP]_i$ (mM)	[b]$[Mg^{2+}]_i$ (µM)	Cs-glutamate (mM)	NaCl (mM)	Cs-HEPES (mM)	Cs-EGTA (mM)	Cs-EDTA (mM)	Mg·ATP (mM)	$MgCl_2$ (mM)
0.0	250	140	8	10	10	3	-	0.38
1.5	250	120	8	10	10	3	2.15	2.71
3.0	250	120	8	10	10	3	4.30	2.10
6.0	250	120	8	10	10	3	8.55	0.85
9.0	250	120	8	10	10	3	12.40	-

[a,b]$[Mg \cdot ATP]_i$ and $[Mg^{2+}]_i$ were calculated using WebMaxC software.

Suppl. Figure S1

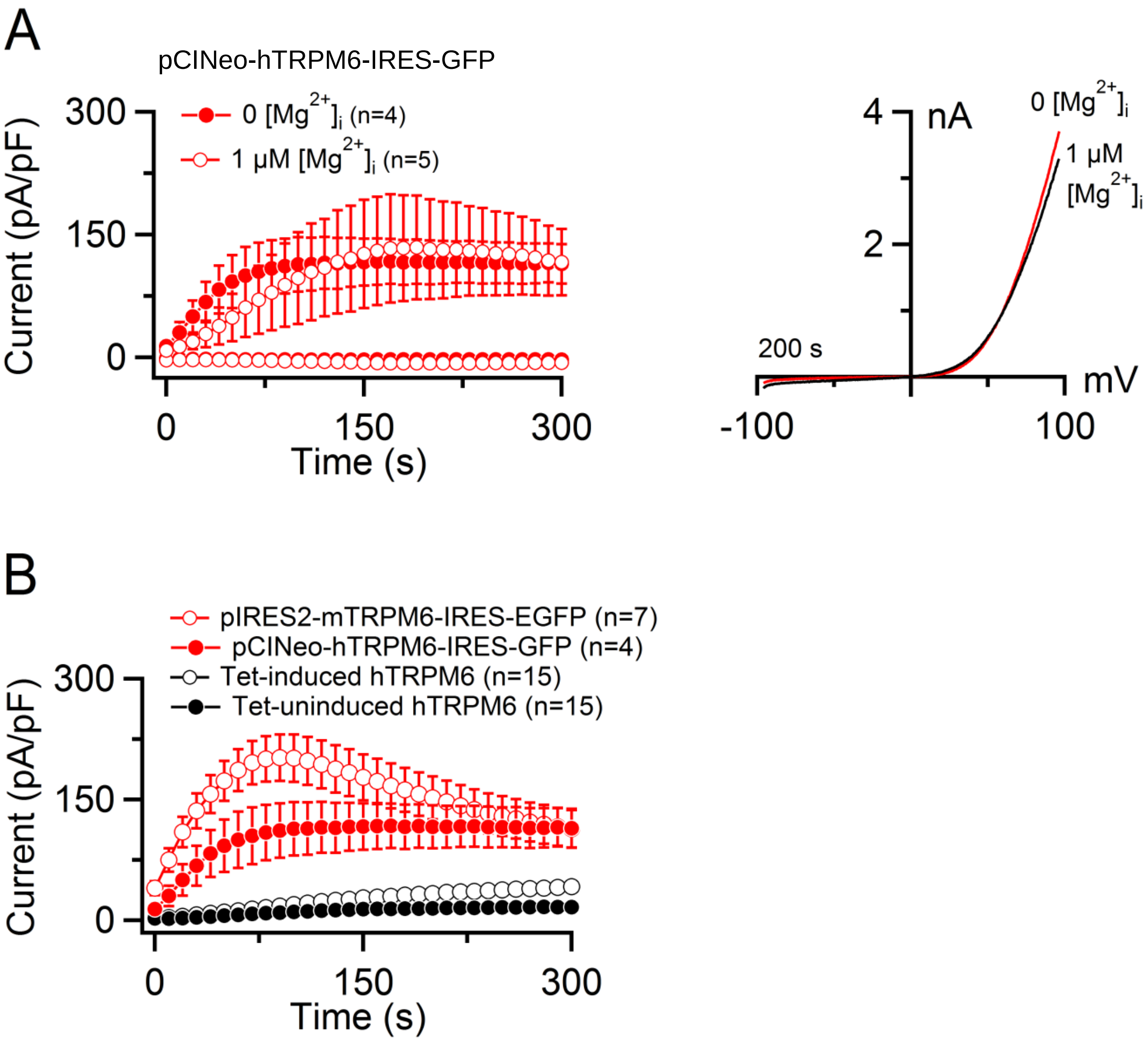

Suppl. Figure S2

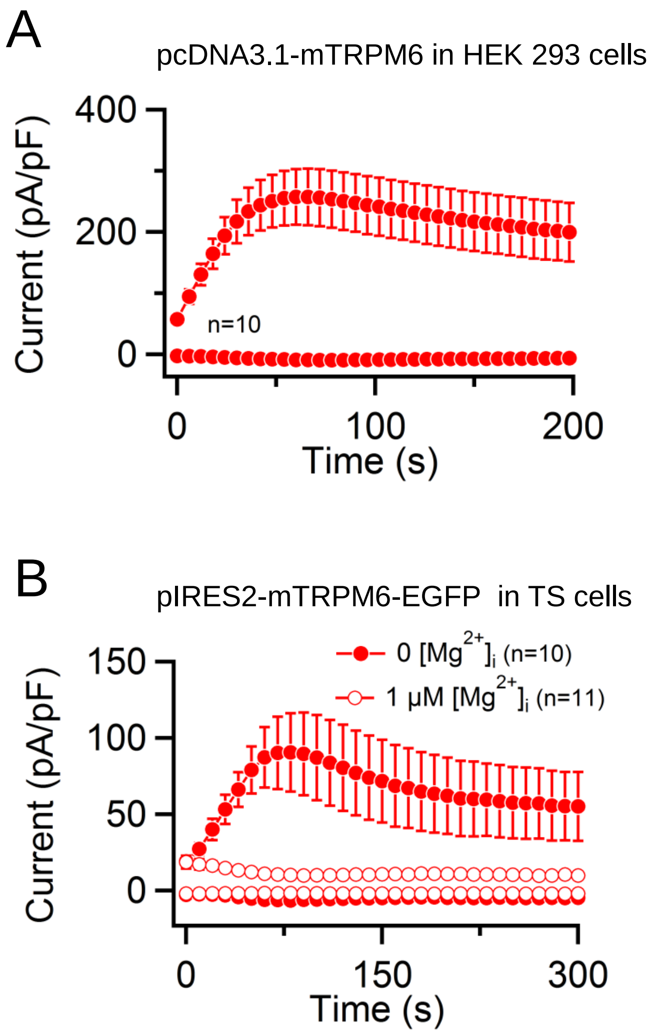

Suppl. Figure S3

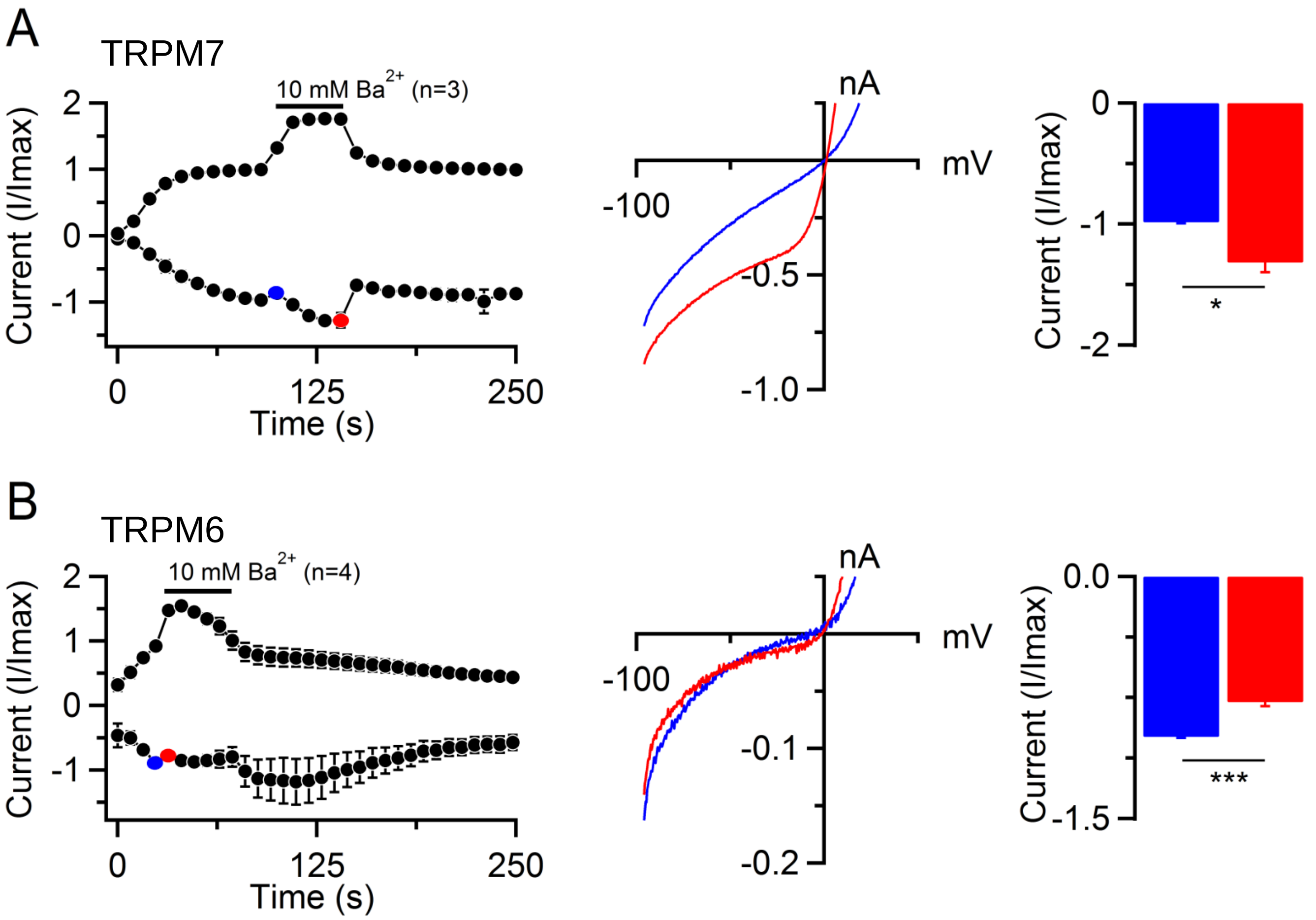

Suppl. Figure S4

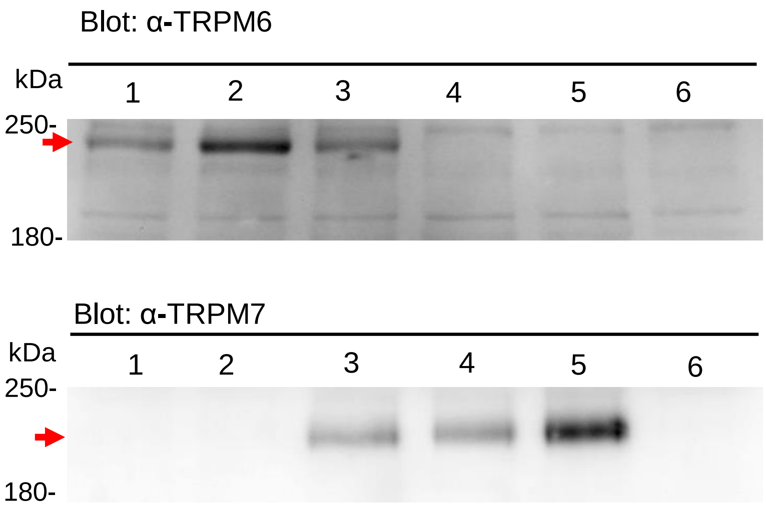

Samples key:

1 - mTRPM6 (1 µg)

2 - mTRPM6 (2 µg)

3 - mTRPM6 (1 µg) + mTRPM7 (1 µg)

4 - mTRPM7 (1 µg)

5 - mTRPM7 (2 µg)

6 - Untransfected cells

Suppl. Figure S5

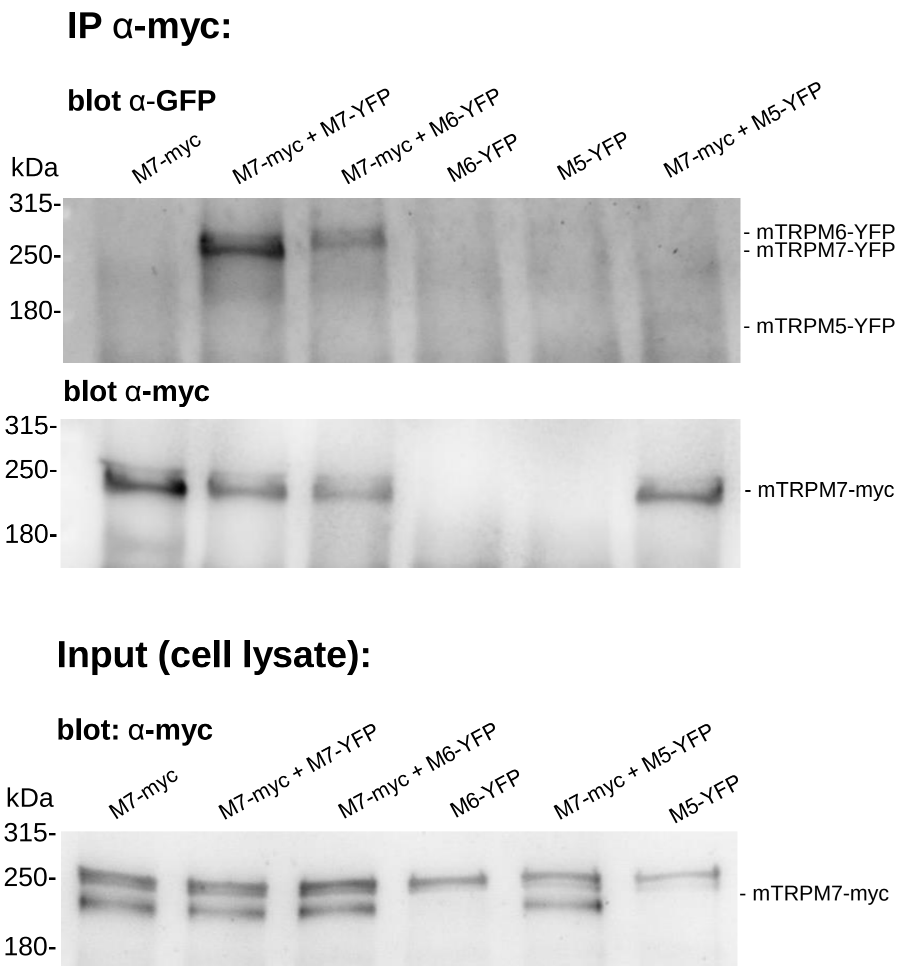

Suppl. Figure S6

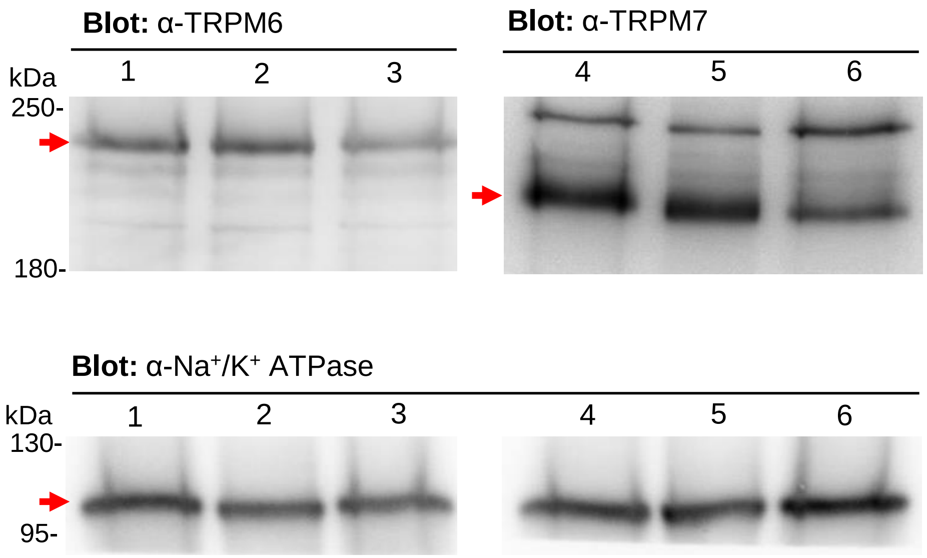

Samples key:

1 - mTRPM6 (20 µg)

2 - mTRPM6 (10 µg)

3 - mTRPM6 (10 µg) + mTRPM7 (10 µg)

4 - mTRPM7 (20 µg)

5 - mTRPM7 (10 µg)

6 - mTRPM6 (10 µg) + mTRPM7 (10 µg)

Suppl. Figure S7

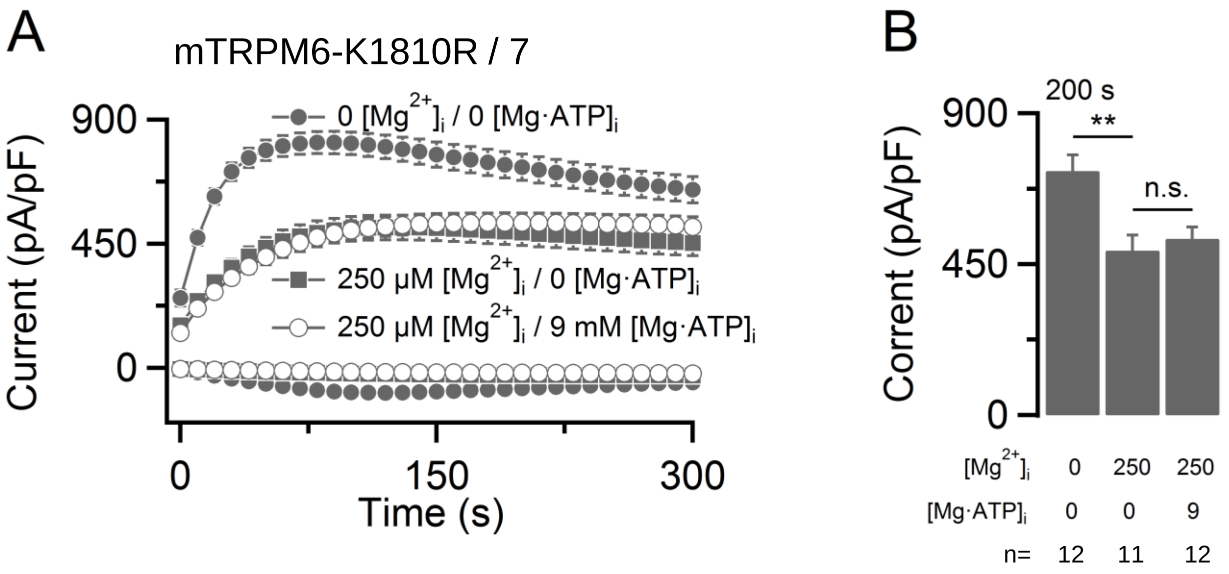

Suppl. Figure S8

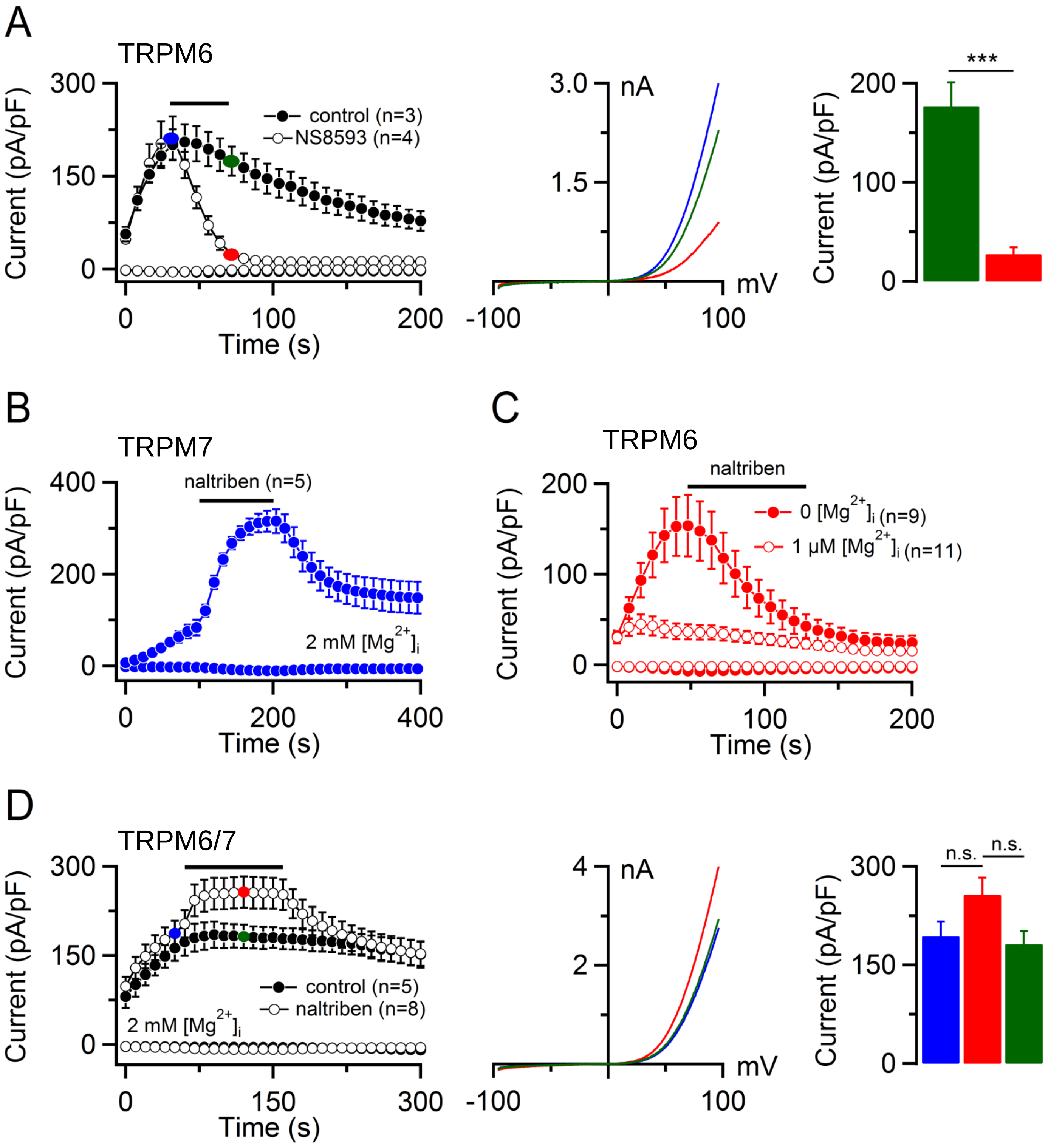

Suppl. Figure S9

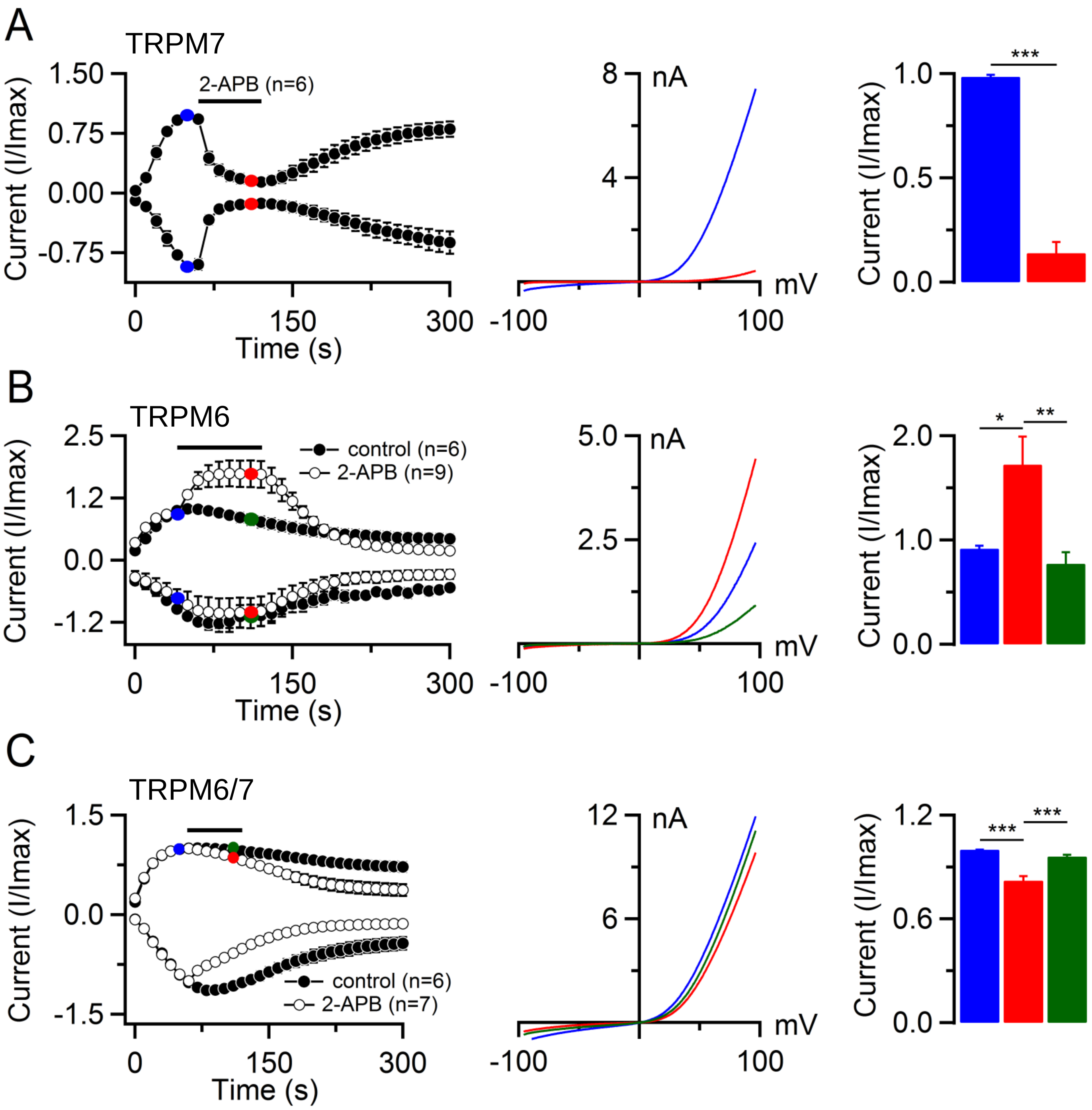

Supplement: Supplementary file 1 — Supplementary information and figures [file 41598_2017_8144_MOESM1_ESM.pdf]
